# Cytotoxic lesions of the corpus callosum: a systematic review

**DOI:** 10.1007/s00330-023-10524-3

**Published:** 2023-12-26

**Authors:** Selina Moors, Dominik Nakhostin, Dariya Ilchenko, Zsolt Kulcsar, Jay Starkey, Sebastian Winklhofer, Benjamin V. Ineichen

**Affiliations:** 1 Department of Neuroradiology, Clinical Neuroscience Center, University Hospital of Zurich, Zurich, Switzerland; 2 Department of Radiology, Oregon Health & Science University, Portland, OR USA; 3 Center for Reproducible Science, University of Zurich, Zurich, Switzerland

**Keywords:** Corpus callosum, Systematic review, Magnetic resonance imaging, Central nervous system diseases, COVID-19

## Abstract

**Objectives:**

Cytotoxic lesions of the corpus callosum (CLOCC) are a common magnetic resonance imaging (MRI) finding associated with various systemic diseases including COVID-19. Although an increasing number of such cases is reported in the literature, there is a lack of systematic evidence summarizing the etiology and neuroimaging findings of these lesions. Thus, the aim of this systematic review was to synthesize the applied nomenclature, neuroimaging and clinical features, and differential diagnoses as well as associated disease entities of CLOCC.

**Materials and methods:**

A comprehensive literature search in three biomedical databases identified 441 references, out of which 324 were eligible for a narrative summary including a total of 1353 patients.

**Results:**

Our PRISMA-conform systematic review identifies a broad panel of disease entities which are associated with CLOCC, among them toxic/drug-treatment-associated, infectious (viral, bacterial), vascular, metabolic, traumatic, and neoplastic entities in both adult and pediatric individuals. On MRI, CLOCC show typical high T2 signal, low T1 signal, restricted diffusion, and lack of contrast enhancement. The majority of the lesions were reversible within the follow-up period (median follow-up 3 weeks). Interestingly, even though CLOCC were mostly associated with symptoms of the underlying disease, in exceptional cases, CLOCC were associated with callosal neurological symptoms. Of note, employed nomenclature for CLOCC was highly inconsistent.

**Conclusions:**

Our study provides high-level evidence for clinical and imaging features of CLOCC as well as associated disease entities.

**Clinical relevance statement:**

Our study provides high-level evidence on MRI features of CLOCC as well as a comprehensive list of disease entities potentially associated with CLOCC. Together, this will facilitate rigorous diagnostic workup of suspected CLOCC cases.

**Key Points:**

*• Cytotoxic lesions of the corpus callosum (CLOCC) are a frequent MRI feature associated with various systemic diseases.*

*• Cytotoxic lesions of the corpus callosum show a highly homogenous MRI presentation and temporal dynamics.*

*• This comprehensive overview will benefit (neuro)radiologists during diagnostic workup.*

**Supplementary Information:**

The online version contains supplementary material available at 10.1007/s00330-023-10524-3.

## Introduction

Transient magnetic resonance imaging (MRI) signal alterations of the corpus callosum are an increasingly discussed topic in neuroradiology [1]. They most frequently manifest as small round or oval lesions in or close to the midline of the splenium of the corpus callosum and are characterized by T2-weighted (w) hyperintensity without gadolinium enhancement [2]. They also commonly show decreased diffusivity [3], potentially because complex cell-cytokine interactions result in water influx into neurons resulting in cytotoxic edema [4].

These callosal lesions are associated with a broad variety of etiologies [5], such as viral or bacterial infections, drug-related, malignancies, or metabolic disorders [4]. Yet, despite the recent increase in interest in these lesions, the exact pathophysiology, their etiology, and their MRI features are not fully understood.

It is also noteworthy to acknowledge a high degree of inconsistency in employed nomenclature. These splenial lesions have been referred to as mild encephalitis/encephalopathy with reversible splenial lesion (MERS) or reversible splenial lesion syndrome (RESLES), or simply as transient splenial lesions (TSL). More recently, the term CLOCC (cytotoxic lesions of the corpus callosum) has been introduced by Starkey and colleagues to more objectively describe this lesions, as the lesions are not always strictly splenial, not always reversible, and not always associated with mild encephalopathy [3]. In our systematic review, we will refer to these lesions as CLOCC, with a discussion of nomenclature at the end of the paper.

Based on these shortcomings, the aim of this study is to perform a systematic review in patients with CLOCC to enhance the understanding of the imaging features and give a more detailed overview of this finding to facilitate diagnostic workup of suspected cases. In particular, we aim at answering the following questions: (1) What are the MRI features of CLOCC? (2) What is the currently used nomenclature for CLOCC including differences between terms and how commonly are these different terms used? (3) What is the imaging differential diagnosis of CLOCC? (4) What are underlying disease entities causing CLOCC? (5) What is the temporal evolution of CLOCC, that is do they change over time and how long does it persist? (6) What is the histopathological correlation of CLOCC? (7) Do patients with CLOCC present with neurological symptoms associated with corpus callosum lesions?

## Materials and methods

We registered the study protocol in the International prospective register of systematic reviews (PROSPERO, CRD42022296487, https://www.crd.york.ac.uk/PROSPERO/) and used the Preferred Reporting Items for Systematic Reviews and Meta-Analysis (PRISMA) Guidelines for reporting [6].

### Search strategy

We searched for original observational studies published in full up to April 12, 2023, in Medline via PubMed, Web of Science, and Ovid EMBASE. See Supplementary Methods for the search string in each of these data bases.

### Inclusion and exclusion criteria

We included all original publications including case reports that reported on cytotoxic lesions of the corpus callosum, as assessed by MRI, and their associations with any human disease. Exclusion criteria: animal studies, non-English articles, and papers which did not include quantitative data. Reviews were excluded but retained as source for additional references.

### Study selection and data extraction

Titles and abstracts of studies were screened for their relevance in the web-based application Rayyan by two reviewers (S.M. and B.V.I.) [7] followed by full-text screening (S.M. and D.N.). Discrepancies were resolved by discussion.

### Quality assessment

The quality of each study was assessed against pre-defined criteria by two reviewers using an adjusted version of the Newcastle–Ottawa scale [8].

## Results

### Eligible publications and general study characteristics

**Fig. 1:**
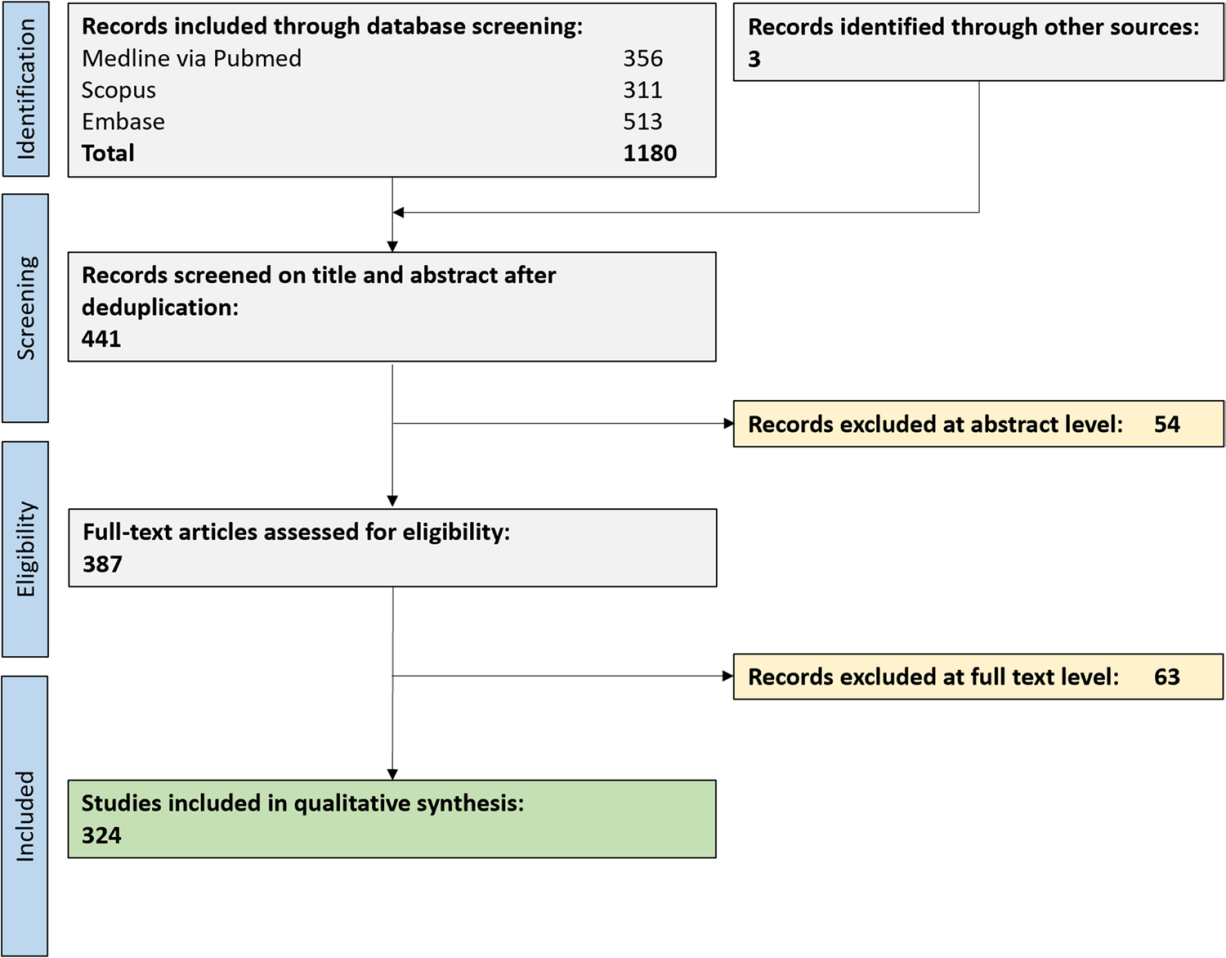
Flow chart depicting the study selection process

In total, 441 unique publications were retrieved from our comprehensive data base search. After full text screening, 324 publications were eligible for this systematic review (Fig. 1).

A total of 189 publications included adult subjects only, comprising a total of 416 patients (217 males [52%], 196 females [47%], three with unknown sex [1%]). Their median age was 37 years (interquartile range, IQR 24–46). One hundred twenty-five publications included children only, comprising a total of 937 children. Their median age was 5.1 years (IQR 4–12).

### Study quality

Most publications (92%) showed a low risk of bias for the selection domain (that is, whether callosal lesions were defined according to acknowledged diagnostic criteria); see Supplementary Table 1. Many studies did not report on adjusting their statistical analyses for subject age, sex, or other potential confounders (comparability domain), thus potentially inducing biases.

### Used nomenclature for callosal lesions

Out of the 324 publications, the most frequently used terms were MERS (mild encephalitis/encephalopathy with reversible splenial lesion) (134 publications, 41%), followed by the term reversible or transient splenial lesions (44, 14%), and RESLES (reversible splenial lesion syndrome) (41, 13%). CLOCC(s) (“Cytotoxic lesions of the corpus callosum”) was used in 26 publications (8%); this term was first proposed by Starkey and colleagues in 2017 [3].

### Imaging/signal characteristics of callosal lesions

Splenial lesions had a highly homogenous MRI presentation (Table 1): they mostly presented with hyperintense signal on T2w/T2w-FLAIR (95%) and hypo- or isointense signal on T1w (70% hypointense, 27% isointense). In addition, the majority of lesions did show restricted diffusion (99%) but absent gadolinium enhancement (98%).

**Table 1:** MRI Imaging characteristics of cytotoxic lesions of the corpus callosum (CLOCC)

T1w	70% hypointense (72) 27% isointense (28) 3% hyperintense (3)
T2w	0% hypointense (0) 5% isointense (10) 95% hyperintense (185)
T2w-FLAIR	0% hypointense (0) 4% isointense (7) 96% hyperintense (156)
DWI	> 99% restricted (274, one case unrestricted)
T1w post contrast (gadolinium)	98% without enhancement (84) 2% with enhancement (2)

The number of publications reporting this finding is listed in brackets

*DWI* diffusion-weighted imaging, *FLAIR* fluid-attenuated inversion recovery

### Location of callosal lesions

CLOCC almost always involved the splenium of the corpus callosum (310/311 publications, > 99%) except in one case with callosal hypogenesis in which only the genu was affected [9]. In cases with more than one discrete location of involvement in the corpus callosum, the additional locations were most commonly the genu in 15 cases (5%), followed by the entire corpus callosum in six cases (2%) and the body in two cases (< 1%). There was no reported involvement of the rostrum of the corpus callosum. Extra-splenial callosal lesions predominantly affected children. Notably, lesions in the genu of the corpus callosum were found mainly in the pediatric population (13 of the 15 cases being children; median age, 8.5 years). Lesions spanning the entire corpus callosum were exclusively observed in children (*n* = 6 children). Only one child and one adult exhibited involvement in the body of the corpus callosum. There was no association of sex or disease entity with CLOCC location (Supplementary Table 4).

### Temporal evolution of callosal lesions

In 283 of 324 (87%) publications, a follow-up MRI was performed. The follow-up time point ranged from a few days to several months with a median interval of 3 weeks (IQR 2–5 weeks).

Most publications (260/283 publications, 92%) reported a complete resolution of the lesion within the follow-up period. Of the 22 follow-up exams which were done after exactly 1 week, 19 (86%) showed a complete resolution. However, in some patients, CLOCC did not show complete resolution (*n* = 25; 9%); one case exhibited a persisting lesion as long as 10 months [10]. Of the persisting or residual cases, 22 were in adults and five in children. Rarely, CLOCC persisted without major changes (number of patients = 2; 1%) until their follow-up at 1 month [2] and 3 months [11], respectively.

### Associated disease classes and entities

A variety of overarching pathology classes were associated with callosal lesions. Most common overarching classes in adults were drug/toxin-induced (including withdrawal of drugs) (26%, commonly by antiepileptic drugs), viral infections (18%, SARS-CoV-2, influenza, and others), (cerebro)vascular diseases (18%, mostly subarachnoid hemorrhage), bacterial infections (10%), and seizures (6%). In 20% of the adult patients, no associated disease could be identified. In children (< 18 years old), there was not a clear association with a disease entity in 42 subjects. Among the identified disease entities, callosal lesions were most frequently associated with viral infections (73%, mostly influenza and rotavirus). Bacterial infections (7%, mostly mycoplasma), seizures, and metabolic entities (3% each) were less common. A summary of associated disease classes and entities can be found in Table 2 for adults and Table 3 for children. For a complete list of associated disease entities, see Supplementary Table 2 for adults and Supplementary Table 3 for children.

**Table 2:** Entities associated with cytotoxic lesions of the corpus callosum (CLOCC) in adults

Drug or drug withdrawal or toxins (*n* = 88) (27%)	Antiepileptic (*n* = 32) Neuroleptic (*n* = 12) Carbamazepine (*n* = 8) Chemotherapy (*n* = 8) Dietary supplement (*n* = 6) Metronidazole (*n* = 6) Immunomodulary drugs (*n* = 4) Toxins (*n* = 4) Other (*n* = 6)
Vascular (*n* = 60) (18%)	Subarachnoid hemorrhage (*n* = 49) Stroke (*n* = 1) Other (*n* = 10)
Viral (*n* = 59) (18%)	COVID-19 (*n* = 15) Influenza virus (*n* = 13) Dengue virus (*n* = 5) Epstein-Barr virus (*n* = 4) Other (*n* = 22)
Bacteria and plasmodia (*n* = 34) (10%)	*Plasmodium falciparum* (*n* = 8) *Mycoplasma pneumoniae* (*n* = 7) *Staphlyococcus aureus* (*n* = 4) other (*n* = 15)
Seizure and epilepsy (*n* = 20) (6%)	Status epilepticus (*n* = 1) Other (*n* = 19)
Metabolic (*n* = 10) (3%)	Hypo- and hyperglycemia (*n* = 10)
Pregnancy-associated (*n* = 8) (2%)	Eclampsia (*n* = 1) other (*n* = 7)
Autoimmune (*n* = 6) (2%)	GFAP antibodies (*n* = 2) Anti-NMDA encephalitis (*n* = 1) SLE (*n* = 1) Other (*n* = 2)
Traumatic (*n* = 4) (1%)	Head trauma (*n* = 4)
Neoplasia (*n* = 4) (1%)	Insulinoma (*n* = 1) Leukemia (*n* = 1) Mantle cell lymphoma (*n* = 1) Melanocytoma (*n* = 1)
Various causes (*n* = 37) (11%)	Alcoholism (*n* = 1) COVID-19 vaccine (*n* = 1) Deep brain stimulation (*n* = 1) High-altitude sickness (*n* = 1) Migraine (*n* = 1) Parkinsonism (*n* = 1) Other (*n* = 31)

In 330 adult patients, an associated disease was identified; in 86 patients, no associated disease entity could be identified (number of subjects per associated disease is reported in brackets). Only diseases which occurred more than once or deemed of special interest are listed in this table, for a detailed list see supplementary data

*Anti-NMDA encephalitis* anti-N-methyl-d-aspartate-receptor encephalitis, *GFAP antibodies* glial fibrillary acidic protein antibodies, *SLE* systemic lupus erythematosus

**Table 3:** Entities associated with cytotoxic lesions of the corpus callosum (CLOCC) in children

Viral (*n* = 409) (75%)	Influenza virus (A or B) (*n* = 187) Rotavirus (*n* = 140) HHV6 (*n* = 16) Adenovirus (*n* = 10) COVID 19 (*n* = 10) Mumps (*n* = 8) Respiratory-syncytial virus (*n* = 6) Epstein-Barr virus (*n* = 4) Viral gastroenteritis unspecified (*n* = 4) Other (*n* = 24)
Bacterial (*n* = 40) (7%)	*Mycoplasma pneumoniae* (*n* = 15) *Enterococcus faecalis* (*n* = 5) *Escheria coli* (*n* = 4) Other (*n* = 16)
Seizure or epilepsy (*n* = 19) (4%)	Benign infantile epilepsy (*n* = 2) Other (*n* = 17)
Electrolyte disbalance (*n* = 18) (3%)	Hyponatremia (*n* = 18)
Vascular (*n* = 13) (2%)	Kawasaki disease (*n* = 13)
Drugs, toxins, and vaccination (*n* = 12) (2%)	Mumps vaccination (*n* = 6) other (*n* = 6)
Traumatic (*n* = 10) (2%)	Diffuse axonal injury (*n* = 9) Unspecified (*n* = 1)
Autoimmune (*n* = 4) (1%)	Anti-GFAP encephalitis (*n* = 2) Systematic lupus erythematosus (*n* = 2)
Various causes (*n* = 17) (3%)	Acute encelopathy in congenital adrenal hyperplasia (*n* = 3) Thyroid crisis (*n* = 2) other (*n* = 12)

In 542 pediatric patients, an associated disease was identified; in 395 patients, no associated disease entity could be identified (number of subjects per associated disease is reported in brackets). Only diseases which occurred more than once or are of special interest are listed in this table; for a detailed list, see the supplementary data

*GFAP antibodies* glial fibrillary acidic protein antibodies, *HHV6* human herpes virus 6, *SLE* systemic lupus erythematosus

### Callosal lesions and associated symptoms

In all but one publication, no neurological symptoms were described with tropism to the corpus callosum but rather symptoms of the associated disease entity. However, intriguingly, one single publication reported on one patient having a callosal lesion and clinically presenting with an alien hand syndrome [12].

### MRI-histopathology correlation

Our systematic literature did not identify studies with MRI-histopathology correlation for callosal lesions.

## Discussion

### Main findings

Here, we systematically assessed MRI features of callosal lesions including their associated clinical disease entities and temporal evolution as well as their nomenclature. Callosal lesions show very homogenous MR signal characteristics: a typical lesion is T1w hypointense, T2w(-FLAIR) hyperintense, and exhibits restricted diffusion but not gadolinium enhancement. These lesions almost ubiquitously affect the splenium of the corpus callosum and are often transient in nature; i.e., they regress within one to few weeks. These lesions are associated with a broad class of disease entities including toxic/drug-treatment-associated, infectious (viral, bacterial), vascular, metabolic, traumatic, and neoplastic (Table 1). No studies were identified which assessed the tissue signature of these callosal lesions.

### Findings in the context of existing evidence

Our systematic review identifies several used terms to denote callosal lesions. Early studies have most commonly used the terms MERS (“mild encephalopathy with reversible lesion in the splenium”) [13] or RESLES (“reversible splenial lesion syndrome”) [14]. In 2017, Starkey and colleagues proposed the term CLOCC (“cytotoxic lesions of the corpus callosum”) [3], mostly because these lesions are not strictly splenial, lesions are not always reversible, and the encephalopathy can also be more severe. Although some recent studies have adopted this nomenclature [15], some studies have retained prior terminology [16]. Some studies defined further subclassifications with small-type lesions isolated to the splenium and large-type lesions spread along the ventricles [17]. It is also noteworthy that the terms used to denote these lesions are sometimes not used interchangeably, with RESLES describing the radiological lesion and MERS the clinically mild course of infectious encephalitis with neurological symptoms like seizures and altered consciousness within the spectrum of RESLES [18, 19].

**Fig. 2:**
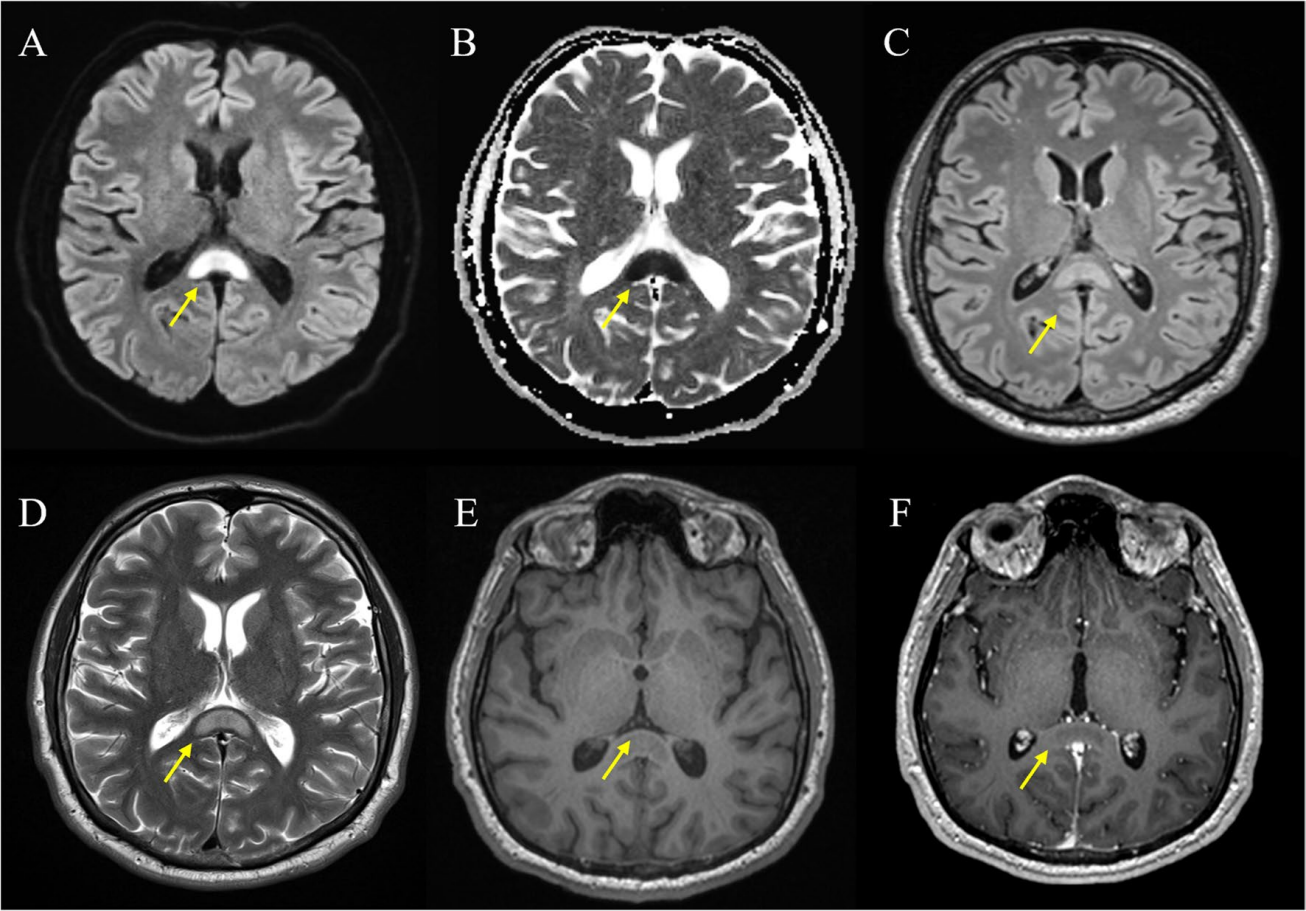
Typical structural MRI findings in cytotoxic lesions of the corpus callosum (CLOCC). Pictorial magnetic resonance imaging (MRI) examples of a 38-year-old patient who presents with a typical cytotoxic lesion of the corpus callosum (CLOCC) after head trauma. **A** DWI B0, oval hyperintensity throughout the splenium and into the adjacent hemispheres (“boomerang sign”). **B** ADC hypointensity due to restricted diffusion.** C** T2w-FLAIR, high signal. **D** T2 slightly hyperintense. **E** T1 native, pre-gadolinium uptake, shows a slight hypointensity in the region. **F** T1 post-gadolinium uptake, no enhancement. Abbreviations. DWI, diffusion-weighted imaging; FLAIR, fluid-attenuated inversion recovery

Our systematic review corroborates the typical and mostly homogenous MRI appearance of callosal lesions: hypointensity on T1w, hyperintensity on T2w, and restricted diffusion (Fig. 2). The few exceptions reported could be reflective of different stages of lesion evolution, underlying pathophysiological mechanisms, or individual patient factors. The lack of gadolinium enhancement is consistent with the cytotoxic, rather than vasogenic, nature of the edema typically associated with these lesions. Although MRI has a limited specificity of underlying tissue pathology, these characteristics are in line with a focal callosal edema. In fact, it has been speculated that a cytotoxic edema is the correlating tissue pathology of splenial lesions [20–22] (reviewed in [3]). The hypothesis suggests a cytokinopathy causing excitotoxicity resulting in an intracellular edema and hence restricted diffusion [23, 24]. This pathological cascade seems to preferentially affect the splenium of the corpus callosum, with its high density of glutamatergic and other excitatory amino acid receptors [25–27]. Even though this is a plausible hypothesis, our systematic review did not identify a single study with MRI-histopathology correlation for splenial lesions. Hence, histopathological validation of callosal lesions is warranted to investigate its actual tissue substrate. Alternatively, advanced MRI methods including diffusion tensor imaging, MR spectroscopy [28], or more specific myelin imaging approaches [29–31] could be harnessed to gain additional insight into pathophysiology of these lesions.

Besides their homogenous MRI presentation, the vast majority of CLOCC (1) does affect the splenium of the corpus callosum and (2) is reversible within 1 to few weeks. In the majority of persisting cases, the follow-up period was notably brief, spanning few weeks; thus, it is possible that the lesion may have resolved in a subsequent period. In the rare instances where the lesion remains evident after several months, one must consider if a different etiology than CLOCC is at play. Biopsies of persistent CLOCC cases could provide further insights into the histopathology of CLOCC potentially distinguishing it from other etiologies.

It is also noteworthy that CLOCC were not described as being associated with specific corpus callosum symptoms, though in one case the CLOCC was associated with alien hand syndrome [12], which has been described before as being caused by lesions in the corpus callosum [32]. Additionally, none of the studies reported advanced neuropsychological testing which could pick up more subtle deficits. Advanced neuropsychological testing could be harnessed to gain additional insight into the neurological manifestations of these lesions.

Our systematic review identified several disease classes and entities which are associated with CLOCC, among them drug-induced (including withdrawal), viral infections, cerebrovascular diseases, and seizures. However, the exact etiology of splenial lesions is still under debate [14]. It has been hypothesized that trauma, infection, or inflammation may cause a macrophage-driven cytokinopathy; this in term would result in glutamatergic excitotoxicity leading to cytotoxic edema in the corpus callosum [3]. Although this pathogenic cascade seems plausible for neuroinflammatory or local traumatic insults, additional yet unknown pathomechanisms could be involved in this cascade for peripheral inflammatory diseases caused by, e.g., rotavirus or systemic bacterial infections. An as of yet to be defined unifying etiology is also possible, analogous to dentate nucleus T1w hyperintensity being attributable to various etiologies was eventually proven to be related to multiple administrations of gadolinium [33].

In diagnostic routine, CLOCC can be misdiagnosed (Table 4). Differential diagnostic entities include other diseases with common callosal/splenial involvement [5, 34, 35], among them (1) ischemic lesions (caused by rare distal occlusion of the anterior cerebral artery); (2) neuroinflammatory conditions such as acute disseminated encephalomyelitis (ADEM) or multiple sclerosis (MS) [36]; (3) neoplastic lesions such as lymphoma, glioblastoma, or metastases; and (4) Marchiafava–Bignami disease, induced by alcohol-related toxic effects on the brain [37]. Other, rare differential diagnoses include atypical presentations of posterior reversible encephalopathy syndrome (PRES), hypoxic-ischemic encephalopathy mainly in children, and changes in patients with hypoglycemic encephalopathy. Of note, CLOCC lesions do not show pathological signals in susceptibility-weighted imaging (SWI) sequences, unlike differential diagnoses such as post-traumatic or neoplastic changes of the corpus callosum [38–40]. Although differential diagnostic workup may be difficult, our systematic review emphasizes the mostly homogenous imaging presentation of CLOCC. In Table 4, we summarize the most common differential diagnosis including their detailed MRI characteristics. With this, a routine clinical MRI protocol is normally sufficient to discriminate splenial lesions from common imaging mimics. Advanced imaging techniques, such as quantitative susceptibility mapping (QSM), might be further helpful to differentiate among various entities, by measuring the concentration and distribution of substances that affect the magnetic susceptibility of tissues, such as iron, myelin, or calcium; however, no systematic analyses have been published to date.

**Table 4:** Common differential diagnoses of cytotoxic lesions of the corpus callosum (CLOCC) including their magnetic resonance imaging (MRI) characteristics

	T1w	T2w-FLAIR	T1w Gd	SWI	DWI	Comments
CLOCC	Hypointense	Hyperintense	No enhancement	No SWI artifacts	Diffusion restriction	Mostly reversible within a few weeks
Lymphoma, glioblastoma, metastases	Usually hypointense	Usually hyperintense	Gd enhancement in the majority of cases	Potential intratumoral susceptibility signals or hermorrhagic components	Diffusion restriction depends on the tumor cellularity	Often in line with a mass effect
Ischemic stroke	Hypointense	Demarcated ischemic tissue hyperintense	Acute: No enhancement Subacute: Gd enhancement Chronic: No enhancement	Usually no SWI artifacts, however hemorrhagic transformation possible	Diffusion restriction in acute and subacute cases	T2w-FLAIR hyperintensity not reversible
MS, ADEM	Hypointense	Hyperintense	Gd enhancement in active lesions	No SWI artifacts	Diffusion restriction might be visible in active lesions	Additional white matter lesions
Diffuse axonal injury (DAI) of the corpus callosum	Hypointense	Hyperintense	No enhancement	Blood products visible in SWI	Diffusion restriction might be visible in acute lesions	Associated with traumatic brain injury and indicates a poor prognosis
Marchiafava-Bignami disease	Hypointense	Usually hyperintense	No enhancement	No SWI artifacts	Diffusion restriction	Usually affects the central layers of the corpus callosum. Lesions resolve after successful therapy

*Abbreviations*: *ADEM* acute demyelinating encephalomyelitis, *DWI* diffusion-weighted imaging, *FLAIR* fluid-attenuated inversion recovery, *Gd* gadolinium, *MS* multiple sclerosis, *SWI* susceptibility-weighted imaging

### Limitations

First, there was considerable heterogeneity in the applied methodology for MRI of callosal lesions which could skew the interpretation of the narrative synthesis. Second, although our study does identify a comprehensive panel of disease entities associated with callosal lesions, no inference about causality can be made based on our analysis. However, of note, also primary studies were not able to discern direction of causality. Third, unsurprisingly, none of the included studies did histopathologically confirm the callosal lesions. Thus, callosal lesions might have been misinterpreted and erroneously included in our study. However, we did mitigate this by ascertaining study quality by acknowledged risk of bias assessment tools.

## Conclusions

Our systematic review provides high level evidence for the homogenous MRI appearance of CLOCC as well as a comprehensive list of associated disease entities. Future research warrants histopathological validation of CLOCC as well as more and larger longitudinal cohorts to investigate causality of disease entities with CLOCC.

### Supplementary Information

Below is the link to the electronic supplementary material.

Supplementary file1 (PDF 261 KB)

## Abbreviations

ADEM: Acute disseminated encephalomyelitis
CLOCC: Cytotoxic lesions of the corpus callosum
FLAIR: Fluid-attenuated inversion recovery
MERS: Mild encephalitis/encephalopathy with reversible splenial lesion
MRI: Magnetic resonance imaging
MS: Multiple sclerosis
RESLES: Reversible splenial lesion syndrome
TSL: Transient splenial lesions

## Declarations

### Guarantor

The scientific guarantor of this publication is Benjamin V. Ineichen.

### Statistics and biometry

No complex statistical methods were necessary for this paper.

### Study subjects or cohorts overlap

No study subjects or cohorts overlap have been previously reported.

### Methodology

• multicenter study
