# Supplementary material for: Cytotoxic lesions of the corpus callosum: a systematic review
Source: Eur Radiol. 2023 Dec 26;34(7):4628–37. doi: 10.1007/s00330-023-10524-3 (PMC11213749; doi:10.1007/s00330-023-10524-3)
Supplement: Supplementary file 1 — Supplementary file1 (PDF 261 KB) [file 330_2023_10524_MOESM1_ESM.pdf]

Supplementary data

Supplementary Table 1: Risk of bias assessment.

First author	Year	S1	S2	S3	S4	C1	C2	O1	O2	O3
Aksu Uzunhan	2021	1	1	NA	NA	1	1	NA	1	NA
Chen	2016	1	1	1	NA	1	1	NA	1	1
Fang	2017	1	1	NA	NA	1	1	NA	1	1
Gao	2020	1	1	NA	NA	1	1	NA	1	1
Gürtler	2005	1	1	1	1	1	1	0	0	1
Hoshino	2012	1	1	NA	NA	1	1	NA	0	0
Kasai.	2020	0	1	NA	NA	1	1	NA	0	0
Kashiwagi	2021	1	1	NA	NA	1	1	NA	1	1
Kashiwagi	2015	1	1	0	1	1	0	0	1	0
Kashiwagi	2014	1	1	NA	NA	1	1	0	1	0
Kato	2011	1	1	1	1	1	1	0	0	0
Kawashima	2012	0	1	NA	NA	1	1	0	0	0
Maruyama	2020	1	1	NA	NA	1	1	NA	0	0
Moteki	2021	1	1	NA	NA	1	1	NA	1	1
Ogawa	2020	1	0	NA	NA	1	1	NA	1	1
Qing	2020	1	1	NA	NA	1	1	NA	1	1
Tada	2004	1	1	NA	NA	1	1	NA	1	1
Takanashi	2009	1	1	0	0	0	0	0	1	0
Takanashi	2009	1	1	1	1	1	1	NA	1	0
Toi	2021	1	1	1	1	0	0	1	1	0
Tsubouchi	2018	1	1	1	1	1	1	0	1	0
Xue	2021	1	1	NA	NA	1	1	NA	1	1
Zhang	2020	1	1	NA	NA	1	1	NA	1	1
Zhu	2016	1	1	NA	NA	1	1	NA	1	1
Zhuang	2019	1	1	NA	NA	1	1	NA	1	1

Risk of bias assessment for publications with more than 10 cases. Selection domain: S1: is the case definition adequate; S2: Representativeness of cases; S3: Selection of controls; S4: Definition of controls; Comparability domain: C1: Sex; C2: Age; Outcome domain: O1: Blinded assessors; O2: Follow-up (1 month or resolution); O3: Drop out (reported or > 70%). Ones indicating low risk of bias, zeros indicating high risk of bias.

Abbreviations: NA, not applicable.

Supplementary Table 2: Complete list of entities associated with cytotoxic lesions of the corpus callosum (CLOCC) in adults.

Drug or drug withdrawal or toxins (n=88) (27%)	Antiepileptic drug (unspecified, n=24)<br>Carbamazepine (n=8)<br>Dietary supplement (n=6)<br>Metronidazole (n=6)<br>5-fluorouracile (n=5)<br>Lithium (n=3)<br>Levetiracetam (n=2)<br>Neuroleptic unspecified (n=2)<br>Neuroleptic malignant syndrome (n=2)<br>Olanzapine (n=2)<br>Phenytoin (n=2)<br>Valproate (n=2)<br>Acute toxic encelopathy unspecified (n=1)<br>Amanita phalloides (n=1)<br>Aripiprazole (n=1)<br>Benzhexol (n=1)<br>Cabergoline (n=1)<br>Capecitabine (n=1)<br>Chemotherapy (n=1)<br>Clozapine (n=1)<br>Corticosteroids (n=1)<br>Dichlormethane (n=1)<br>Diuretic (n=1)<br>Glyophosphat poisoning (n=1)<br>Indomethacine (n=1)<br>Ipilimumab (n=1)<br>IvIg (n=1)<br>Lorazepam (n=1)<br>Mannitol (n=1)<br>Metothrexate (n=1)<br>Oxcarbamazepine (n=1)<br>Risperidone (n=1)<br>Tegafur uracil (n=1)<br>Trihexyphenidyl (n=1)<br>Vigabatrin (n=1)
Viral (n=59) (18%)	COVID 19 (n=15)<br>Influenza virus(n=13)<br>Dengue virus (n=5)<br>Eppstein-barr virus (n=4)<br>Adenovirus (n=2)<br>Hepatitis A virus (n=2)<br>Puumala hanta virus (n=2)<br>Rotavirus (n=2)<br>Viral encephalitis unspecified (n=2)<br>Chikungunya virus (n=1)<br>Cytomegalovirus (n=1)<br>HHV6 (n=1)<br>HIV (n=1)<br>Japanese encephalitis virus (n=1)

	Measles (n=1)<br>Mumps (n=1)<br>Severe fever with thrombomozytopenia virus (n=1)<br>Tick-borne encephalitis (n=1)<br>Varicella zoster virus (n=1)<br>Viral meningitis unspecified (n=1)<br>Viral respiratory tract infection (n=1)
Vascular (n=60) (18%)	Subarachnoidal hemorrhage (n=49)<br>Cerebral venous thrombosis (n=2)<br>Ischemic stroke (n=2)<br>Fat embolism in sickle cell disease (n=1)<br>Pulmonary embolism (n=1)<br>Reversible cerebral vasoconstriction syndrome (n=1)<br>Segmental artery mediolysis (n=1)<br>Subdural empyema (n=1)<br>Subdural hematoma surgery (n=1)<br>Thrombotic thrombocytopenic purpura (n=1)
Bacterial and plasmodia (n=34) (10%)	Plasmodium falciparum (n=8)<br>Mycoplasma pneumoniae (n=7)<br>Staphlyococcus aureus (n=4)<br>Legionella (n=3)<br>Salmonella typhii (n=2)<br>Bortadella pertussis (n=1)<br>Chlamydia pneumoniae (n=1)<br>Enterococcus faecalis (n=1)<br>Escheria coli (n=1)<br>Klebsiella pneumoniae (n=1)<br>Meningococcus (n=1)<br>Mycobacterium tuberculosis (n=1)<br>Orientia tsutsugamushi (n=1)<br>Staphylococcus aureus (n=1)<br>Streptococcus pneumoniae (n=1)
Seizure and epilepsy (n=20) (6%)	Epilepsy (n=17)<br>Seizure (n=2)<br>Status epilepticus (n=1)
Metabolic (n=10) (3%)	Hypoglycemia (n=7)<br>Acyl-CoA dehydrogenase deficiency (n=1)<br>Diabetes (n=1)<br>Hyperglycemia (n=1)
Pregnancy-associated (n=8) (2%)	Eclampsia (n=1)<br>Eclampsia-associated PRES (n=1)<br>Postpartum (n=1)<br>Postpartum cerebral angiopathy (n=1)<br>Postpartum encelopathy (n=1)<br>Postpartum preeclampsia (n=1)<br>Postpartum psychosis (n=1)<br>Pregnancy (n=1)

Autoimmune (n=6) (1.8%)	GFAP antibodies (n=2)<br>Anti-VGKC antibodies (n=1)<br>Anti-yo rhombencephalitis (n=1)<br>NMDA encephalitis (n=1)<br>SLE (n=1)
Trauma (n=4) (1.2%)	Traumatic brain injury (n=4)
Neoplasia (n=4) (1.2%)	Insulinoma (n=1)<br>Leucemia (n=1)<br>Mantle cell lymphoma (n=1)<br>Melanocytoma (n=1)
Other (38) (12%)	Demyelination disease (n=3)<br>Hyponatremia (n=3)<br>Marchiafava-Bignami Disease (n=3)<br>Csf pressure drop (n=2)<br>Acute intermittent porphyria (n=1)<br>Acute onset psychosis (n=1)<br>Acute urinary retention (n=1)<br>Acyl-CoA dehydrogenase deficiency (n=1)<br>Alcoholism (n=1)<br>Aseptic meningomyelitis (n=1)<br>Atypical HUS (n=1)<br>Blood transfusion (n=1)<br>Cesareansection (n=1)<br>COVID vaccine (n=1)<br>Deep brain stimulation (n=1)<br>Delirious mania (n=1)<br>ECMO (n=1)<br>Fanconi syndrome (n=1)<br>Gastric bypass (n=1)<br>Graves disease (n=1)<br>HaNDL (n=1)<br>Hemicrania continua (n=1)<br>High altitude sickness (n=1)<br>Hyperthyroidism (n=1)<br>Hypernatremia (n=1)<br>Hypoxia (n=1)<br>Meningitis retention syndrome (n=1)<br>Migraine (n=1)<br>Parkinsonism (n=1)<br>Rabies vaccine (n=1)<br>Thyroid storm (n=1)

In 330 patients an associated disease was identified, in 86 patients, no associated disease could be identified (number of subjects per associated disease is reported in brackets).

Abbreviations: CSF, cerebrospinal fluid; ECMO, extracorporeal membrane oxygenation; GFAP, Glial fibrillary acidic protein; HaNDL, headache accompanied with transient neurologic deficits and cerebrospinal fluid lymphocytosis; HHV-6, human herpes virus 6; HIV, human immunodeficiency virus; HUS, Hemolytic-uremic syndrome; IVIg, intravenous immunoglobulins; NMDA, N-methyl-D-

aspartate; PRES, Posterior Reversible Encephalopathy Syndrome; SLE, systemic lupus erythematosus; VGKC, voltage-gated potassium channel.

Supplementary table 3: Complete list of entities associated with cytotoxic lesions of the corpus callosum (CLOCC) in children.

Viral (n=409) (75.5%)	Influenza virus (A or B) (n=187)<br>Rotavirus (n=140)<br>Adenovirus (n=10)<br>Human herpes virus 6 (n=16)<br>COVID 19 (n=10)<br>Mumps (n=8)<br>Respiratory-syncytial virus (n=6)<br>Eppstein-barr-virus (n=4)<br>Viral gastroenteritis unspecified (n=4)<br>Coxsackie Virus (n=3)<br>Cytomegalovirus (n=3)<br>Echovirus (n=3)<br>Herpes-simplex virus (n=3)<br>Viral encelopathy, unspecified (n=2)<br>Viral respiratory infection, unspecified (n=2)<br>Parvovirus B19 (n=2)<br>Varizella-zoster virus (n=2)<br>Dengue virus (n=1)<br>Hand-foot-mouth disease (n=1)<br>Parainfluenza virus (n=1)<br>Rhinovirus (n=1)
Bacterial (40) (7.4%)	Mycoplasma pneumoniae (n=15)<br>Enterococcus faecalis (n=5)<br>Escheria coli (n=4)<br>Streptococcus pneumoniae (n=3)<br>Listeria monocytogenes (n=2)<br>Salmonella (n=2)<br>Bacterial encelopathy (n=1)<br>Bacterial menigitis (n=1)<br>Cryptococcus (n=1)<br>Group B streptococcus (n=1)<br>Klebsiella pneumoniae (n=1)<br>Legionella (n=1)<br>Meningococcemia (n=1)<br>Neisseria meningitidis (n=1)<br>Rickettsia rickettsii (n=1)
Seizure or epilepsy (n=19) (3.5%)	Epilepsy (n=10)<br>Seizure (n=7)<br>Benign infantile epilepsy (n=2)
Electrolyte disbalance (n=18) (3.3%)	Hyponatremia (n=18)
Vascular (n=13) (2.4%)	Kawasaki Disease (n=13)
Drug, toxine and vaccination (12) (2.2%)	Mumps vaccination (n=6)<br>Antiepileptica (n=1)<br>Carbon monoxide (n=1)

	Electronic-cigarette or vaping product use-associated lung injury EVALI (n=1)<br>Steroids (n=1)<br>Sulfonylurea Intoxikation (n=1)<br>Tetracycline (n=1)
Trauma (n=10) (1.8%)	DAI (n=9)<br>Brain injury (n=1)
Autoimmune (n=4) (0.7%)	Anti GFAP astrocytopathy (n=2)<br>SLE (n=2)
Other (17) (3.1%)	Acute encelopathy in congenital adrenal hyperplasia (n=3)<br>Thyroid crisis (n=2)<br>Acute toxic encelopathy unspecified cause (n=1)<br>Adrenocortical insufficiency (n=1)<br>Apparent-life threatening event in infancy (n=1)<br>acute lymphoblastic leukemia (n=1)<br>Argininosuccinic adicuria (n=1)<br>Hyperglycemia (n=1)<br>Hypothermia (n=1)<br>Hypoglycemia (n=1)<br>Mitochondrial disease (n=1)<br>Saposin B deficiency (n=1)<br>Status migrainosus (n=1)<br>Stool amoeba cysts (n=1)

In 542 patients an associated disease was identified, in 395 patients the cause remained unknown (number of subjects per associated disease is reported in brackets).

Abbreviations: *DAI, diffuse axonal injury; GFAP, glial fibrillary acidic protein; SLE, systemic lupus erythematosus.*

Supplementary table 4: Complete list of entities, sex, and age associated with lesions other than splenium.

Location involving	Male sex (%)	Disease entity	Age
Genu (n=15)	77%	Mycoplasma pneumoniae (n=3)<br>Benign partial epilepsy in infancy (n=2)<br>Influenza A (n=2)<br>Influenza unspecified (n=1)<br>Adenovirus (n=1)<br>Non alcoholic partially reversible Marchiafava-Bignami Disease (n=1)<br>Respiratory syncytial virus (n=1)<br>Kawasaki Disease (n=1)<br>Rotavirus enterocolitis (n=1)<br>Unknown (n=2)	13 children (median age 8.5 years)<br>2 adults (median age 52 years)
Corpus (n=2)	(50%)	Non alcoholic partially reversible Marchiafava-Bignami Disease (n=1)<br>Influenza A (n=1)	1 adult (78 years)<br>1 child (10 years)
Entire (n=6)	(50%)	Parainfluenza (n=1)<br>Adenovirus (n=1)<br>Salmonella enteritis (n=1)<br>Influenza A (n=1)<br>e. coli pyelonephritis (n=1)<br>Unknown (n=1)	6 children (median age 8 years)

Search string

(Cytotoxic lesion* of the corpus callosum) OR (cloccs) OR (transient lesion* of the splenium of the corpus callosum) OR (transient focal lesion* in the splenium of the corpus callosum) OR (transient splenial lesion*) OR (mild encephalitis with a reversible isolated SCC lesion) OR (mild encephalopathy with a reversible isolated SCC lesion) OR (mild encephalopathy with reversible splenial lesions) OR (reversible splenial lesion*) OR ("reversible splenial lesion syndrome") OR (RESLES) OR (clinically silent lesion* in the splenium of the corpus callosum)

List of publications included in the systematic review (in alphabetical order)

1. Abenhaim Halpern, L., et al., Mild encephalopathy with splenial lesion and parainfluenza virus infection. Pediatr Neurol, 2013. 48(3): p. 252-4.

2. Achalia, R. and C. Andrade, Reversible abnormality of the splenium in a bipolar patient with neuroleptic malignant syndrome. Bipolar Disord, 2014. 16(7): p. 773-5.

3. Akbar, A. and S. Ahmad, Atypical case of mild encephalopathy/encephalitis with reversible splenial lesion of the corpus callosum (MERS) associated with Mycoplasma pneumoniae infection in a paediatric patient. BMJ Case Rep, 2021. 14(8).

4. Akın, M.E., et al., Mild encephalopathy with reversible extensive white matter lesions in a child with acute adenoviral infection and a literature review. Turk J Pediatr, 2021. 63(3): p. 516-521.

5. Aksu, B., et al., Reversible corpus callosum splenial lesion due to steroid therapy. J Neuroimaging, 2015. 25(3): p. 501-4.

6. Aksu Uzunhan, T., et al., Cytotoxic lesions of the corpus callosum in children: Etiology, clinical and radiological features, and prognosis. Brain Dev, 2021. 43(9): p. 919-930.

7. Al-Awwad, A.A. and A. Koriesh, Cytotoxic Lesion in the Splenium of Corpus Callosum Secondary to Subacute Methotrexate Neurotoxicity. Avicenna J Med, 2021. 11(3): p. 160-162.

8. Al-Edrus, S., et al., Reversible splenial lesion syndrome in neuroleptic malignant syndrome. Biomed Imaging Interv J, 2009. 5(4): p. e24.

9. Alakbarova, N., et al., Mild encephalitis/encephalopathy with a reversible splenial lesion (MERS) development after Amanita phalloides intoxication. Acta Neurol Belg, 2016. 116(2): p. 211-3.

10. Altunkas, A., et al., MRI findings of a postpartum patient with reversible splenial lesion syndrome (RESLES). Acta Neurol Belg, 2016. 116(3): p. 347-9.

11. Anada, R., et al., [A case of clinically mild encephalitis/encephalopathy with a reversible splenial lesion (MERS) associated with infectious endocarditis caused by Staphylococcus aureus]. Rinsho Shinkeigaku, 2019. 59(10): p. 666-668.

12. Anneken, K., et al., Transient lesion in the splenium related to antiepileptic drug: case report and new pathophysiological insights. Seizure, 2008. 17(7): p. 654-7.

13. Appenzeller, S., et al., Focal transient lesions of the corpus callosum in systemic lupus erythematosus. Clin Rheumatol, 2006. 25(4): p. 568-71.

14. Arakawa, C., et al., Detection of group a rotavirus RNA and antigens in serum and cerebrospinal fluid from two children with clinically mild encephalopathy with a reversible splenial lesion. Jpn J Infect Dis, 2011. 64(3): p. 204-7.

15. Aslan, K., et al., Reversible Focal Splenial Lesion on Diffusion-Weighted MRI in Sulfonylurea Intoxication. J Belg Soc Radiol, 2015. 99(1): p. 34-36.

16. Avcu, G., et al., Mild encephalitis/encephalopathy with reversible splenial lesion (MERS) associated with Streptococcus pneumoniae Bacteraemia. J Infect Public Health, 2017. 10(4): p. 479-482.
17. Azuma, J., et al., Marked elevation of urinary β2-microglobulin in patients with reversible splenial lesions: A small case series. J Neurol Sci, 2016. 368: p. 109-12.
18. Bagatti, D. and G. Messina, Cytotoxic Lesion in the Splenium of Corpus Callosum Associated with Intracranial Infection After Deep Brain Stimulation. World Neurosurg, 2020. 135: p. 306-307.
19. Bektaş, G., N. Akçay, K. Boydağ, and E. Şevketoğlu, Reversible splenial lesion syndrome associated with SARS-CoV-2 infection in two children. Brain Dev, 2021. 43(2): p. 230-233.
20. Bellani, M., et al., Adult Mild Encephalitis With Reversible Splenial Lesion Associated With Delirious Mania: A Case Report. Front Psychiatry, 2020. 11: p. 79.
21. Bulakbasi, N., M. Kocaoglu, C. Tayfun, and T. Ucoz, Transient splenial lesion of the corpus callosum in clinically mild influenza-associated encephalitis/encephalopathy. AJNR Am J Neuroradiol, 2006. 27(9): p. 1983-6.
22. Carrara, G., et al., Transient edematous lesions of the splenium in epileptic patients. Can J Neurol Sci, 2005. 32(3): p. 352-5.
23. Caulo, M., et al., Non-alcoholic partially reversible marchiafava-bignami disease: review and relation with reversible splenial lesions. A case report and literature review. Neuroradiol J, 2009. 22(1): p. 35-40.
24. Chauffier, J., et al., [Not Available]. Infect Dis Now, 2021. 51(1): p. 99-101.
25. Chen, W.X., et al., Reversible splenial lesion syndrome in children: Retrospective study and summary of case series. Brain Dev, 2016. 38(10): p. 915-927.
26. Chen, Z., M. Xu, D. Shang, and B. Luo, A case of reversible splenial lesions in late postpartum preeclampsia. Intern Med, 2012. 51(7): p. 787-90.
27. Cho, J.S., et al., Mild encephalopathy with reversible lesion in the splenium of the corpus callosum and bilateral frontal white matter. J Clin Neurol, 2007. 3(1): p. 53-6.
28. Chopra, P., R.S. Bhatia, and R. Chopra, Mild Encephalopathy/Encephalitis with Reversible Splenial Lesion in a Patient with Salmonella typhi Infection: An Unusual Presentation with Excellent Prognosis. Indian J Crit Care Med, 2019. 23(12): p. 584-586.
29. Chougar, L., et al., Retrospective Observational Study of Brain MRI Findings in Patients with Acute SARS-CoV-2 Infection and Neurologic Manifestations. Radiology, 2020. 297(3): p. E313-e323.
30. Cirnigliaro, G., et al., Treatment-related transient splenial lesion of the Corpus Callosum in patients with neuropsychiatric disorders: a literature overview with a case report. Expert Opin Drug Saf, 2020. 19(3): p. 315-325.
31. Cohen-Gadol, A.A., et al., Transient postictal magnetic resonance imaging abnormality of the corpus callosum in a patient with epilepsy. Case report and review of the literature. J Neurosurg, 2002. 97(3): p. 714-7.

32. Conry, R.M., J.C. Sullivan, and L.B. Nabors, 3rd, Ipilimumab-induced encephalopathy with a reversible splenial lesion. Cancer Immunol Res, 2015. 3(6): p. 598-601.

33. Conti, M., et al., Transient focal lesion in the splenium of the corpus callosum: MR imaging with an attempt to clinical-physiopathological explanation and review of the literature. Radiol Med, 2007. 112(6): p. 921-35.

34. Cortese, R., et al., Reversible splenial lesion and complex visual disturbances due to carbamazepine withdrawal. Neurol Sci, 2015. 36(8): p. 1515-6.

35. Cursi, L., et al., HHV6-related mild encephalopathy with reversible splenial lesion (MERS) presenting with urinary and fecal retention in an Italian adolescent. Infez Med, 2019. 27(1): p. 97-102.

36. da Rocha, A.J., et al., Focal transient lesion in the splenium of the corpus callosum in three non-epileptic patients. Neuroradiology, 2006. 48(10): p. 731-5.

37. Dabaja, E., R. Daoud, O. Alquadan, and J.Y. Ang, A Case of a Rare Tick-Borne Illness in Michigan Associated With Reversible Splenial Lesion Syndrome: When It Is Not a Viral Exanthem. Clin Pediatr (Phila), 2020. 59(9-10): p. 938-942.

38. Dadak, M., et al., Varying Patterns of CNS Imaging in Influenza A Encephalopathy in Childhood. Clin Neuroradiol, 2020. 30(2): p. 243-249.

39. Daniel, E., A.K. Mishra, P. Mannam, and I. Ramya, Reversible splenial lesion in primary dengue fever. J Vector Borne Dis, 2019. 56(3): p. 272-275.

40. Dar, M.E., G. McInerney, and S. Pridmore, Reversible splenial lesion of the corpus callosum. Australas Psychiatry, 2008. 16(1): p. 55-6.

41. De Greef, J., C. Jaumotte, B. Quivron, and G. Derue, Reversible splenial lesion in auto-immune thyroid disease: a case report. Acta Clin Belg, 2014. 69(3): p. 208-9.

42. Degirmenci, E., T. Degirmenci, E.N. Cetin, and Y. Kıroğlu, Mild encephalitis/encephalopathy with a reversible splenial lesion (MERS) in a patient presenting with papilledema. Acta Neurol Belg, 2015. 115(2): p. 153-5.

43. Dogan, V.B., et al., Transient lesion in splenium of the corpus callosum presenting as mild encephalopathy. Neurol Sci, 2018. 39(4): p. 769-771.

44. Doherty, M.J., et al., Clinically mild encephalitis/encephalopathy with a reversible splenial lesion. Neurology, 2005. 64(8): p. 1487; author reply 1487.

45. Dong, K., et al., Mild encephalopathy with a reversible splenial lesion mimicking transient ischemic attack: A case report. Medicine (Baltimore), 2016. 95(44): p. e5258.

46. Dong, X. and S. Cong, Reversible splenial lesion syndrome associated with acute Mycoplasma pneumoniae-associated encephalitis: A report of four cases and literature review. Exp Ther Med, 2018. 16(3): p. 2152-2159.

47. Duberkar, D. and R. Jawale, Transient lesion in the splenium of corpus callosum due to abrupt phenytoin withdrawal. Neurol India, 2017. 65(Supplement): p. S104.

48. Edjlali, M., et al., Teaching NeuroImages: Cytotoxic lesions of the corpus callosum in encephalopathic patients with COVID-19. Neurology, 2020. 95(22): p. 1021-1022.

49. Eguchi, K., K. Tsuzaka, I. Yabe, and H. Sasaki, Reversible splenial lesion in new-onset refractory status epilepticus: A case report. Clin Neurol Neurosurg, 2019. 183: p. 105392.

50. Ehler, E., et al., Exposure to iodomethane and dichloromethane associated with a confusional state. Neurotoxicology, 2011. 32(3): p. 307-11.

51. El Aoud, S., et al., A first case of Mild Encephalitis with Reversible Splenial Lesion(MERS) as a presenting feature of SARS-CoV-2. Rev Neurol (Paris), 2021. 177(1-2): p. 139-141.

52. Elkhaled, W., et al., A 23-Year-Old Man with SARS-CoV-2 Infection Who Presented with Auditory Hallucinations and Imaging Findings of Cytotoxic Lesions of the Corpus Callosum (CLOCC). Am J Case Rep, 2020. 21: p. e928798.

53. Faa, D.E.O., D.E.M. Tfb, and P.A.S. Rocha-Filho, Transient lesion in the splenium of the corpus callosum associated with COVID-19. Arq Neuropsiquiatr, 2020. 78(11): p. 738.

54. Fang, Q., et al., Clinically mild encephalitis/encephalopathy with a reversible splenial lesion of corpus callosum in Chinese children. Brain Dev, 2017. 39(4): p. 321-326.

55. Feraco, P., et al., Mild Encephalitis/Encephalopathy with Reversible Splenial Lesion (MERS) due to Cytomegalovirus: Case Report and Review of the Literature. Neuropediatrics, 2018. 49(1): p. 68-71.

56. Fluss, J., et al., Mild influenza-associated encephalopathy/encephalitis with a reversible splenial lesion in a Caucasian child with additional cerebellar features. Eur J Paediatr Neurol, 2010. 14(1): p. 97-100.

57. Fong, C.Y., et al., Mild encephalitis/encephalopathy with reversible splenial lesion (MERS) due to dengue virus. J Clin Neurosci, 2017. 36: p. 73-75.

58. Forestier, G., I. de Beaurepaire, G. Bornet, and G. Boulouis, Cytotoxic lesion of the corpus callosum as presenting neuroradiological manifestation of COVID-2019 infection. J Neurol, 2021. 268(5): p. 1595-1597.

59. Fu, M.L., N. Han, and W. Wang, Cytomegalovirus-Associated Mild Encephalopathy/Encephalitis With Reversible Splenial Lesion. Neurologist, 2021. 26(5): p. 172-174.

60. Fuchigami, T., et al., A 4-year-old girl with clinically mild encephalopathy with a reversible splenial lesion associated with rotavirus infection. J Infect Chemother, 2013. 19(1): p. 149-53.

61. Fukagawa, K., M. Izumi, K. Higuchi, and Y. Adachi, Reversible splenial lesion associated with Staphylococcus aureus endocarditis. Intern Med, 2013. 52(10): p. 1147-8.

62. Fukuda, S., et al., Rotavirus-associated encephalopathy with a reversible splenial lesion. Pediatr Neurol, 2009. 40(2): p. 131-3.

63. Fuseya, Y., K. Komatsu, and S. Matsumoto, Transient Splenial Lesion Following Abrupt Withdrawal of Carbamazepine. Intern Med, 2017. 56(8): p. 989-990.

64. Galnares-Olalde, J.A., et al., Cytotoxic Lesions of the Corpus Callosum Caused by Thermogenic Dietary Supplements. AJNR Am J Neuroradiol, 2019. 40(8): p. 1304-1308.
65. Ganapathy, S., E.H. Ey, B.J. Wolfson, and N. Khan, Transient isolated lesion of the splenium associated with clinically mild influenza encephalitis. Pediatr Radiol, 2008. 38(11): p. 1243-5.
66. Gao, X., et al., Clinical analysis of reversible splenial lesion syndrome in Chinese adults: A retrospective study of 11 cases. Medicine (Baltimore), 2020. 99(36): p. e22052.
67. García Fernández, C., P.A. López, A. Caride, and E. Carnero Contentti, Reversible splenial lesion syndrome: A differential diagnosis of corpus callosum lesions. Neurol Psychiatry Brain Res, 2018. 30: p. 99-100.
68. Garcia-Monco, J.C., et al., Reversible splenial lesion syndrome (RESLES): what's in a name? J Neuroimaging, 2011. 21(2): p. e1-14.
69. Gasparini, A., et al., Reversible splenial lesion in neuroleptic malignant syndrome. Panminerva Med, 2018. 60(3): p. 134-135.
70. Gatto, A., et al., Mild encephalitis/encephalopathy with reversible splenial lesion (MERS) in twin sisters with two CD36 frameshift mutations. Neurol Sci, 2020. 41(8): p. 2271-2274.
71. Gaur, P., et al., COVID-19-Associated Cytotoxic Lesions of the Corpus Callosum. AJNR Am J Neuroradiol, 2020. 41(10): p. 1905-1907.
72. Gawlitza, M., K.T. Hoffmann, and D. Lobsien, Mild encephalitis/encephalopathy with reversible splenial and cerebellar lesions (MERS type II) in a patient with hemolytic uremic syndrome (HUS). J Neuroimaging, 2015. 25(1): p. 145-6.
73. Ge, Y.X., Y.Y. Lin, Q.Q. Bi, and Y.J. Chen, Reversible Splenial Lesion Syndrome (RESLES) After Chemotherapy of Oral Tegafur-uracil in a Female With Locally Rectal Adenocarcinoma. Cogn Behav Neurol, 2020. 33(4): p. 283-287.
74. Gellman, S.R. and Y.T. Ng, Transient Corpus Callosal Lesion Presenting with Alien Hand Syndrome. Pediatr Neurol, 2018. 89: p. 66-67.
75. Gilder, T.R., J.S. Hawley, and B.J. Theeler, Association of reversible splenial lesion syndrome (RESLES) with Anti-VGKC autoantibody syndrome: a case report. Neurol Sci, 2016. 37(5): p. 817-9.
76. Göçmen, R. and E. Ünal, Transient splenial lesion in a case with carbon monoxide poisoning: A clue supporting the excitotoxicity hypothesis? Eur J Paediatr Neurol, 2015. 19(6): p. 716-8.
77. Goto, T., et al., Reversible Splenial Lesion Related to Acute Lithium Intoxication in a Bipolar Patient: A Case Report. J Clin Psychopharmacol, 2016. 36(5): p. 528-9.
78. Grosset, L., et al., Mild encephalopathy with reversible splenial lesion: Description of nine cases and review of the literature. Seizure, 2021. 88: p. 83-86.
79. Grühbaum, B., H. Salzer, C. Nasel, and I. Lernbass, Reversible cytotoxic oedema in the splenium of the corpus callosum related to tetracycline therapy. Pediatr Radiol, 2010. 40(10): p. 1693-5.

80. Gunaydin, M. and F. Ozsahin, Transient visual loss: Transient lesion in the splenium of the corpus callosum. Turk J Emerg Med, 2018. 18(3): p. 128-130.
81. Guo, K., et al., Anti-glial fibrillary acidic protein antibodies as a cause of reversible splenial lesion syndrome (RESLES): a case report. Neurol Sci, 2021. 42(9): p. 3903-3907.
82. Guo, Y., et al., Encephalitis with reversible splenial and deep cerebral white matter lesions associated with Epstein-Barr virus infection in adults. Neuropsychiatr Dis Treat, 2017. 13: p. 2085-2092.
83. Gürtler, S., et al., Transient lesion in the splenium of the corpus callosum and antiepileptic drug withdrawal. Neurology, 2005. 65(7): p. 1032-6.
84. Güven, H., S. Delibaş, and S.S. Comoğlu, Transient lesion in the splenium of the corpus callosum due to carbamazepine. Turk Neurosurg, 2008. 18(3): p. 264-70.
85. Hagemann, G., et al., Multiple reversible MR signal changes caused by Epstein-Barr virus encephalitis. AJNR Am J Neuroradiol, 2006. 27(7): p. 1447-9.
86. Han, J., et al., A case of reversible splenial lesion syndrome secondary to Fanconi syndrome with white matter swelling as the main manifestation. J Int Med Res, 2021. 49(1): p. 300060520985713.
87. Hantson, P., D. Hernalsteen, and G. Cosnard, Reversible splenial lesion syndrome in cerebral malaria. J Neuroradiol, 2010. 37(4): p. 243-6.
88. Hara, M., et al., A case of clinically mild encephalitis with a reversible splenial lesion (MERS) after mumps vaccination. Brain Dev, 2011. 33(10): p. 842-4.
89. Hashimoto, Y., et al., A splenial lesion with transiently reduced diffusion in clinically mild encephalitis is not always reversible: A case report. Brain Dev, 2009. 31(9): p. 710-2.
90. Hatanaka, M., et al., Overlapping MERS and mild AESD caused by HHV-6 infection. Brain Dev, 2015. 37(3): p. 334-8.
91. Hayashi, M., et al., COVID-19-associated mild encephalitis/encephalopathy with a reversible splenial lesion. J Neurol Sci, 2020. 415: p. 116941.
92. Hayashi, Y., et al., Reversible splenial lesion of the corpus callosum associated with meningococcal meningitis. J Neurol Sci, 2017. 373: p. 81-82.
93. Hibino, M., et al., Transient hemiparesis and hemianesthesia in an atypical case of adult-onset clinically mild encephalitis/ encephalopathy with a reversible splenial lesion associated with adenovirus infection. Intern Med, 2014. 53(11): p. 1183-5.
94. Hidaka, M., et al., Meningitis retention syndrome associated with complicated mild encephalitis/encephalopathy with reversible splenial lesion in a young adult patient: a case report. Oxf Med Case Reports, 2021. 2021(10): p. omab092.
95. Hiraga, A., K. Koide, Y. Aotsuka, and S. Kuwabara, Reversible Cerebral Vasoconstriction Syndrome with Transient Splenial Lesions after Delivery. Intern Med, 2016. 55(22): p. 3357-3359.
96. Hirayama, Y., Y. Saito, and Y. Maegaki, "Symptomatic" infection-associated acute encephalopathy in children with underlying neurological disorders. Brain Dev, 2017. 39(3): p. 243-247.

97. Honda, K., et al., Transient splenial lesion of the corpus callosum after acute withdrawal of antiepileptic drug: a case report. Magn Reson Med Sci, 2006. 5(4): p. 211-5.

98. Hong, J.M. and I.S. Joo, A case of isolated and transient splenial lesion of the corpus callosum associated with disseminated Staphylococcus aureus infection. J Neurol Sci, 2006. 250(1-2): p. 156-8.

99. Hooshmand, S.I., K. Chow, and A.Z. Obeidat, Teaching NeuroImages: Cytotoxic Lesion of the Corpus Callosum Secondary to Influenza A. Neurology, 2021. 96(12): p. e1697-e1698.

100. Hoshino, A., et al., Epidemiology of acute encephalopathy in Japan, with emphasis on the association of viruses and syndromes. Brain Dev, 2012. 34(5): p. 337-43.

101. Hosoda, A., et al., The first case of recurrent ultra late onset group B streptococcal sepsis in a 3-year-old child. IDCases, 2017. 7: p. 16-18.

102. Howard-Jones, A.R., et al., Mild encephalitis/encephalopathy with reversible splenial lesion in association with Staphylococcus aureus bacteraemia. J Paediatr Child Health, 2021.

103. Hu, S., et al., Thrombotic thrombocytopenic purpura with reversible splenial lesion syndrome: a case report. BMC Neurol, 2020. 20(1): p. 122.

104. Ikeno, M., et al., Gastric perforation and critical illness polyneuropathy after steroid treatment in a patient with encephalitis/encephalopathy with transient splenial lesion. Brain Dev, 2017. 39(4): p. 356-360.

105. Imamura, T., et al., Sisters with clinically mild encephalopathy with a reversible splenial lesion (MERS)-like features; Familial MERS? J Neurol Sci, 2010. 290(1-2): p. 153-6.

106. Imataka, G., et al., MERS associated with bacterial translocation in a pediatric patient with congenital portal vein hypoplasia: A case report. Exp Ther Med, 2018. 16(4): p. 2831-2834.

107. Imataka, G. and S. Yoshihara, Typical MRI Imaging with Clinically Mild Encephalitis/Encephalopathy of a Reversible Splenial Lesion (MERS) Caused by Influenza A Virus. Iran J Public Health, 2020. 49(1): p. 191-192.

108. Issa, N., C. Martin, C. Dulau, and F. Camou, Severe anti-GFAP meningo-encephalomyelitis following viral infection. Mult Scler Relat Disord, 2020. 45: p. 102448.

109. Itamura, S., M. Kamada, and N. Nakagawa, Kawasaki disease complicated with reversible splenial lesion and acute myocarditis. Pediatr Cardiol, 2011. 32(5): p. 696-9.

110. Ito, S., et al., Transient splenial lesion of the corpus callosum in H1N1 influenza virus-associated encephalitis/encephalopathy. Intern Med, 2011. 50(8): p. 915-8.

111. Iwata, A., et al., Reversible splenial lesion associated with novel influenza A (H1N1) viral infection. Pediatr Neurol, 2010. 42(6): p. 447-50.

112. Iype, M., S. Ahamed, B. Thomas, and L. Kailas, Acute encephalopathy with a lesion of the splenium of the corpus callosum--a report of two cases. Brain Dev, 2012. 34(4): p. 322-4.

113. Jang, Y.Y. and K.H. Lee, Transient splenial lesion of the corpus callosum in a case of benign convulsion associated with rotaviral gastroenteritis. Korean J Pediatr, 2010. 53(9): p. 859-62.

114. Jeong, S.H., J.H. Lee, and A.Y. Lee, Reversible visual memory in transient splenial lesion. Neurology, 2009. 73(8): p. 647.

115. Jeong, T.O., et al., Reversible Splenial Lesion Syndrome (RESLES) Following Glufosinate Ammonium Poisoning. J Neuroimaging, 2015. 25(6): p. 1050-2.

116. Jiang, L., S. Mao, J. Xu, and F. Gao, Reversible splenial lesion syndrome in children with benign convulsions associated with mild gastroenteritis: A retrospective study of five cases. Brain Dev, 2019. 41(3): p. 271-275.

117. Jing, C., et al., Reversible splenial lesion syndrome due to oxcarbazepine withdrawal: case report and literature review. J Int Med Res, 2018. 46(3): p. 1277-1281.

118. Ka, A., et al., Mild encephalopathy with reversible splenial lesion: an important differential of encephalitis. Eur J Paediatr Neurol, 2015. 19(3): p. 377-82.

119. Kaino, K., et al., Reversible splenial lesion syndrome with a hyperosmolar hyperglycemic state and neuroleptic malignant syndrome caused by olanzapine. J Diabetes Investig, 2017. 8(3): p. 392-394.

120. Kaminski, J.A. and H. Prüss, N-methyl-d-aspartate receptor encephalitis with a reversible splenial lesion. Eur J Neurol, 2019. 26(6): p. e68-e69.

121. Kaneko, M., et al., Unusual presentation of a severely ill patient having severe fever with thrombocytopenia syndrome: a case report. J Med Case Rep, 2017. 11(1): p. 27.

122. Kaplan, T.B. and A.L. Berkowitz, Reversible splenial lesion syndrome. Pract Neurol, 2016. 16(1): p. 78-9.

123. Karampatsas, K., et al., Rotavirus-associated mild encephalopathy with a reversible splenial lesion (MERS)-case report and review of the literature. BMC Infect Dis, 2015. 15: p. 446.

124. Kasai, M., et al., Epidemiological changes of acute encephalopathy in Japan based on national surveillance for 2014-2017. Brain Dev, 2020. 42(7): p. 508-514.

125. Kashiwagi, M., et al., Reversible splenial lesions during febrile illness with or without white matter lesions. Brain Dev, 2021. 43(6): p. 698-704.

126. Kashiwagi, M., et al., Differential diagnosis of delirious behavior in children with influenza. Brain Dev, 2015. 37(6): p. 618-24.

127. Kashiwagi, M., et al., Clinico-radiological spectrum of reversible splenial lesions in children. Brain Dev, 2014. 36(4): p. 330-6.

128. Kasuga, Y., et al., Acute Focal Bacterial Nephritis Associated With Central Nervous System Manifestations: A Report of 2 Cases and Review of the Literature. Pediatr Emerg Care, 2017. 33(6): p. 418-421.

129. Kato, T., et al., Transient and mild reduction of consciousness during febrile illness in children. Neuropediatrics, 2011. 42(5): p. 183-7.

130. Kawashima, H., et al., National survey of pandemic influenza A (H1N1) 2009-associated encephalopathy in Japanese children. J Med Virol, 2012. 84(8): p. 1151-6.

131. Kim, E., et al., MR imaging of metronidazole-induced encephalopathy: lesion distribution and diffusion-weighted imaging findings. AJNR Am J Neuroradiol, 2007. 28(9): p. 1652-8.
132. Kim, J.H., J.Y. Choi, S.B. Koh, and Y. Lee, Reversible splenial abnormality in hypoglycemic encephalopathy. Neuroradiology, 2007. 49(3): p. 217-22.
133. Kim, T.W., I.S. Park, and J.S. Kim, Reversible splenial lesion following rapid withdrawal of carbamazepine. Can J Neurol Sci, 2014. 41(4): p. 504-5.
134. Kitami, M., et al., Acute urinary retention in a 23-year-old woman with mild encephalopathy with a reversible splenial lesion: a case report. J Med Case Rep, 2011. 5: p. 159.
135. Klironomos, S., et al., Nervous System Involvement in Coronavirus Disease 2019: Results from a Retrospective Consecutive Neuroimaging Cohort. Radiology, 2020. 297(3): p. E324-e334.
136. Ko, S.Y., et al., Reversible splenial lesion on the corpus callosum in nonfulminant hepatitis A presenting as encephalopathy. Clin Mol Hepatol, 2014. 20(4): p. 398-401.
137. Koksel, Y., et al., "CHOICES": An acronym to aid in delineating potential causes of non-metabolic, non-infectious acute toxic leukoencephalopathy. Eur J Radiol Open, 2019. 6: p. 243-257.
138. Kometani, H., et al., Marked elevation of interleukin-6 in mild encephalopathy with a reversible splenial lesion (MERS) associated with acute focal bacterial nephritis caused by Enterococcus faecalis. Brain Dev, 2014. 36(6): p. 551-3.
139. Kosami, K., et al., Clinically mild encephalitis/encephalopathy with a reversible splenial lesion caused by methicillin-sensitive Staphylococcus aureus bacteremia with toxic shock syndrome: a case report. BMC Infect Dis, 2016. 16: p. 160.
140. Kouga, T., et al., A child with three episodes of reversible splenial lesion. Neuropediatrics, 2013. 44(4): p. 199-202.
141. Ku, M.C., et al., Hemorrhagic fever with renal syndrome-related encephalopathy: magnetic resonance imaging findings. Clin Imaging, 2015. 39(6): p. 975-8.
142. Kühn, S., et al., An Observational Cerebral Magnetic Resonance Imaging Study Following 7 Days at 4554 m. High Alt Med Biol, 2019. 20(4): p. 407-416.
143. Kwon, D.Y., et al., Reversible splenial lesion in adult hepatitis A virus associated encephalopathy. Acta Neurol Belg, 2010. 110(2): p. 214.
144. Laizane, G., et al., Rotavirus-associated seizures and reversible corpus callosum lesion. Acta Med Litu, 2019. 26(2): p. 113-117.
145. Landais, A., Reversible splenium diffusion weighted MRI changes associated with hypoglycemia. J Diabetes Complications, 2015. 29(4): p. 607-10.
146. Laothamatas, J., et al., Transient lesion in the splenium of the corpus callosum in acute uncomplicated falciparum malaria. Am J Trop Med Hyg, 2014. 90(6): p. 1117-1123.
147. Le Guennec, L., M. Schmidt, A. Combes, and C.E. Luyt, Reversible splenial lesion syndrome during venoarterial extracorporeal membrane oxygenation. Intensive Care Med, 2019. 45(7): p. 1019-1020.

148. Lebecque, O., N. Mulquin, and M. Dupont, Cytotoxic Lesion of the Corpus Callosum Caused by Puumala Hantavirus Infection. J Belg Soc Radiol, 2019. 103(1): p. 11.
149. Lee, S., et al., Clinical and MRI characteristics of acute encephalopathy in congenital adrenal hyperplasia. J Neurol Sci, 2011. 306(1-2): p. 91-3.
150. Li, C., et al., Reversible splenial lesion syndrome associated with lobar pneumonia: Case report and review of literature. Medicine (Baltimore), 2016. 95(39): p. e4798.
151. Li, J., et al., Is it coincidental or correlative between reversible splenial lesion syndrome and atrial septal defect?: A case report. Medicine (Baltimore), 2020. 99(43): p. e22920.
152. Li, X.F., et al., Clinical Characteristics of H1N1 Influenza A-Associated Mild Encephalopathy with Reversible Splenial Lesion: 4 Pediatric Cases. Curr Med Sci, 2021. 41(4): p. 815-820.
153. Li, X.L., et al., Mild encephalitis/encephalopathy with a reversible splenial lesion associated with respiratory syncytial virus infection in infants. J Neurovirol, 2021. 27(4): p. 638-643.
154. Lin, D. and M. Rheinboldt, Reversible splenial lesions presenting in conjunction with febrile illness: a case series and literature review. Emerg Radiol, 2017. 24(5): p. 599-604.
155. Lin, F.Y. and C.Y. Yang, Reversible splenial lesion of the corpus callosum in migraine with aura. Neurologist, 2011. 17(3): p. 157-9.
156. Lin, J., et al., Cytotoxic Lesion of the Corpus Callosum in an Adolescent with Multisystem Inflammatory Syndrome and SARS-CoV-2 Infection. AJNR Am J Neuroradiol, 2020. 41(11): p. 2017-2019.
157. Lin, Y.J., et al., The Reversible Corpus Callosum Splenium Lesion in A Neonate with Hypoglycemia and Seizure. Acta Neurol Taiwan, 2015. 24(1): p. 15-8.
158. Linden, K., et al., [Transient splenial lesion in influenza A H1N1 2009 infection]. Radiologe, 2011. 51(3): p. 220-2.
159. Liu, J., et al., Reversible splenial lesion syndrome (RESLES) coinciding with cerebral venous thrombosis: a report of two cases. Ther Adv Neurol Disord, 2017. 10(12): p. 375-379.
160. Liu, W.M. and C.H. Lin, A reversible stroke-like splenial lesion in viral encephalopathy. Acta Neurol Taiwan, 2013. 22(3): p. 117-21.
161. Lu, P.L., J.F. Hodes, X. Zheng, and X.Y. Hu, Reversible Splenial Lesion Syndrome with Some Novel Causes and Clinical Manifestations. Intern Med, 2020. 59(20): p. 2471-2480.
162. Ma, X., W. Su, and H. Chen, Reversible splenial lesion syndrome after blood transfusion presents callosal disconnection syndrome: A case report. Medicine (Baltimore), 2018. 97(24): p. e11127.
163. Maeda, M., et al., Transient splenial lesion of the corpus callosum associated with antiepileptic drugs: evaluation by diffusion-weighted MR imaging. Eur Radiol, 2003. 13(8): p. 1902-6.
164. Maeda, M., et al., Reversible splenial lesion with restricted diffusion in a wide spectrum of diseases and conditions. J Neuroradiol, 2006. 33(4): p. 229-36.

165. Maekawa, T., et al., Time-dependent Diffusion in Transient Splenial Lesion: Comparison between Oscillating-gradient Spin-echo Measurements and Monte-Carlo Simulation. Magn Reson Med Sci, 2021. 20(2): p. 227-230.
166. Malhotra, H.S., R.K. Garg, M.R. Vidhate, and P.K. Sharma, Boomerang sign: Clinical significance of transient lesion in splenium of corpus callosum. Ann Indian Acad Neurol, 2012. 15(2): p. 151-7.
167. Man, B.L. and Y.P. Fu, The first case of mild encephalopathy with a reversible splenial lesion due to Japanese encephalitis virus infection. BMJ Case Rep, 2013. 2013.
168. Manappallil, R.G., S.V. Nair, A. Kakkattil, and B. Josphine, Transient splenial lesion due to non-cirrhotic hyperammonaemia in dengue fever. BMJ Case Rep, 2019. 12(6).
169. Manjubashini, D., et al., Magnetic Resonance Imaging in Peripartum Encephalopathy: A Pictorial Review. J Neurosci Rural Pract, 2021. 12(2): p. 402-409.
170. Mao, X.J., B.C. Zhu, T.M. Yu, and G. Yao, Adult severe encephalitis/encephalopathy with a reversible splenial lesion of the corpus callosum: A case report. Medicine (Baltimore), 2018. 97(26): p. e11324.
171. Marsala, S.Z., et al., Mild Encephalitis with a Reversible Splenial Lesion: A Clinical Benign Condition, often Underrecognized - Clinical Case and Literature Review. J Neurosci Rural Pract, 2017. 8(2): p. 281-283.
172. Maruyama, Y., M. Sato, Y. Inaba, and T. Fukuyama, Comparison of mild encephalopathy with reversible splenial lesion with and without acute focal bacterial nephritis. Brain Dev, 2020. 42(1): p. 56-63.
173. Masiello, E., et al., Mild encephalopathy with reversible splenial lesion associated with echovirus 6 infection: a case report and review of the literature. Turk J Pediatr, 2020. 62(2): p. 293-309.
174. Mathew, M., R. Thomas, V. S, and M. Pulicken, Severe Dengue with Rapid Onset Dementia, Apraxia of Speech and Reversible Splenial Lesion. J Neurosci Rural Pract, 2021. 12(3): p. 608-610.
175. Matsubara, K., et al., Reversible splenial lesion in influenza virus encephalopathy. Pediatr Neurol, 2007. 37(6): p. 431-4.
176. Matsubara, K., et al., Thyroid crisis mimicking clinically mild encephalitis/encephalopathy with a reversible splenial lesion: A pediatric case report. Brain Dev, 2021. 43(4): p. 596-600.
177. Matsuo, T., et al., Metronidazole-induced encephalopathy and cytotoxic lesion of the corpus callosum in a patient with diabetic foot infection. Int J Infect Dis, 2019. 89: p. 112-115.
178. Matsuoka, T., et al., A Case of Mild Encephalopathy with a Reversible Splenial Lesion Associated with G5P[6]Rotavirus Infection. Case Rep Pediatr, 2013. 2013: p. 197163.
179. Mawatari, M., et al., Mild encephalitis/encephalopathy with a reversible splenial lesion due to Plasmodium falciparum malaria: a case report. Trop Med Health, 2018. 46: p. 37.
180. Mazur-Melewska, K., et al., Transient lesion in the splenium of the corpus callosum due to rotavirus infection. Childs Nerv Syst, 2015. 31(6): p. 997-1000.

181. McKenzie, E.D., et al., Salmonella Typhi Bacteremia and Mild Encephalitis with a Reversible Splenial Lesion. Can J Neurol Sci, 2020. 47(2): p. 261-263.

182. Melenotte, C., et al., Measles encephalitis the return: mild encephalitis with reversible splenial lesion. Int J Infect Dis, 2013. 17(1): p. e72-3.

183. Merizalde, M., et al., Manic espisode, confusional syndrome and reversible splenial lesion after abrupt withdrawal of oxcarbazepine. J Affect Disord, 2017. 210: p. 122-124.

184. Messina, M.D., T.L. Levin, and E. Blumfield, Cytotoxic lesion of the splenium of the corpus callosum in a patient with EVALI. Clin Imaging, 2020. 66: p. 73-76.

185. Micci, L. and T. DesRosiers, Too Hot to Handle: Early Temperature Management and Unique Treatment of Hyperpyrexia in SARS-CoV2 Encephalopathy. Mil Med, 2021.

186. Mirsattari, S.M., D.H. Lee, M.W. Jones, and W.T. Blume, Transient lesion in the splenium of the corpus callosum in an epileptic patient. Neurology, 2003. 60(11): p. 1838-41.

187. Miyakawa, Y., et al., Agraphia with reversible splenial corpus callosum lesion caused by hypoglycemia. Brain Dev, 2018. 40(7): p. 592-595.

188. Miyata, R., et al., Oxidative stress in patients with clinically mild encephalitis/encephalopathy with a reversible splenial lesion (MERS). Brain Dev, 2012. 34(2): p. 124-7.

189. Mizutani, A.U., et al., Reversible splenial lesion in a patient with new-onset refractory status epilepticus (NORSE). eNeurologicalSci, 2020. 18: p. 100220.

190. Mogi, T., et al., Clinically mild encephalopathy with a reversible splenial lesion and nonconvulsive status epilepticus in a schizophrenic patient with neuroleptic malignant syndrome. Psychiatry Clin Neurosci, 2017. 71(3): p. 212.

191. Mohamed, E., et al., Cerebral malaria with diffuse subcortical microhaemorrhages and a transient splenial lesion. Acta Neurol Belg, 2015. 115(3): p. 399-400.

192. Moreau, A., et al., Cytotoxic lesions of the corpus callosum (CLOCCs) associated with SARS-CoV-2 infection. J Neurol, 2021. 268(5): p. 1592-1594.

193. Mori, H., et al., Reversible splenial lesion in the corpus callosum following rapid withdrawal of carbamazepine after neurosurgical decompression for trigeminal neuralgia. J Clin Neurosci, 2012. 19(8): p. 1182-4.

194. Morichi, S., et al., High production of interleukin-10 and interferon-γ in influenza-associated MERS in the early phase. Pediatr Int, 2012. 54(4): p. 536-8.

195. Morii, K., et al., Reversible splenial lesion of the corpus callosum associated with bacterial meningitis. Int J Infect Dis, 2014. 19: p. 107-8.

196. Moteki, Y., T. Kobayashi, and T. Kawamata, Clinical Significance of Cytotoxic Lesions of the Corpus Callosum in Subarachnoid Hemorrhage Patients: A Retrospective Analysis. Cerebrovasc Dis, 2021. 50(4): p. 405-411.

197. Nagamura, N. and H. Higuchi, Segmental Arterial Mediolysis with Preceding Symptoms Resembling Viral Infection Hampers the Differentiation from Polyarteritis Nodosa. Intern Med, 2019. 58(18): p. 2721-2726.
198. Nagpal, K., P. Agarwal, A. Kumar, and R. Reddi, Chikungunya infection presenting as mild encephalitis with a reversible lesion in the splenium: a case report. J Neurovirol, 2017. 23(3): p. 501-503.
199. Nakajima, M., S. Suda, and K. Kimura, Mild Encephalitis/Encephalopathy with a Reversible Splenial Lesion in an Adult with Cerebellar Ataxia: A Case Report. J Nippon Med Sch, 2020. 87(3): p. 153-156.
200. Nakamura, M., H. Iwasa, and K. Kojima, Central Nervous System Involvement in Mantle Cell Lymphoma Presenting Magnetic Resonance Imaging Features of Mild Encephalitis/Encephalopathy with a Reversible Splenial Lesion. Intern Med, 2021. 60(10): p. 1597-1600.
201. Namatame, C., et al., A thyroid storm patient with protracted disturbance of consciousness and reversible lesion in the splenium of corpus callosum: A case report. Medicine (Baltimore), 2018. 97(7): p. e9949.
202. Narita, H., et al., Transient lesion in the splenium of the corpus callosum, possibly due to carbamazepine. Psychiatry Clin Neurosci, 2003. 57(5): p. 550-1.
203. Natsume, J., et al., Transient splenial lesions in children with "benign convulsions with gastroenteritis". Brain Dev, 2007. 29(8): p. 519-21.
204. Nelles, M., et al., Transient splenium lesions in presurgical epilepsy patients: incidence and pathogenesis. Neuroradiology, 2006. 48(7): p. 443-8.
205. Nojo, T., H. Takao, and J. Kohyama, Simultaneous diffusion-weighted magnetic resonance images and brain blood perfusion scintigraphy for a transient lesion in the splenium of the corpus callosum. Brain Dev, 2008. 30(3): p. 200-2.
206. Notebaert, A., et al., Expanding the spectrum of MERS type 2 lesions, a particular form of encephalitis. Pediatr Neurol, 2013. 48(2): p. 135-8.
207. Nozaki, F., et al., Reversible splenic lesion in a patient with Febrile Infection-Related Epilepsy Syndrome (FIRES). Neuropediatrics, 2013. 44(5): p. 291-4.
208. Numoto, S., H. Kurahashi, H. Iwayama, and A. Okumura, A trial of lacosamide for benign convulsions with gastroenteritis. Brain Dev, 2020. 42(7): p. 551-554.
209. Ogawa, C., et al., Splenial Lesions in Benign Convulsions With Gastroenteritis Associated With Rotavirus Infection. Pediatr Neurol, 2020. 109: p. 79-84.
210. Oger, V., et al., Mild Encephalitis/Encephalopathy with reversible splenial lesion syndrome: An unusual presentation of anti-GFAP astrocytopathy. Eur J Paediatr Neurol, 2020. 26: p. 89-91.
211. Ogul, H., S. Kaya, and Y. Ogul, Transient Splenial Lesion of the Corpus Callosum After Cabergoline Treatment. World Neurosurg, 2018. 114: p. 257-258.

212. Ohashi, E., et al., Transient Probst Bundle Diffusion Restriction: An Acute Encephalopathy Equivalent to Clinically Mild Encephalopathy with a Reversible Splenial Lesion. Intern Med, 2021. 60(16): p. 2667-2670.
213. Ohnishi, T., et al., Apnea and delirious behavior caused by mild encephalitis/encephalopathy with reversible splenial lesion complicated with rotavirus infection. Pediatr Int, 2018. 60(6): p. 602-604.
214. Okada, T., et al., Increased cytokines/chemokines and hyponatremia as a possible cause of clinically mild encephalitis/encephalopathy with a reversible splenial lesion associated with acute focal bacterial nephritis. Brain Dev, 2021.
215. Okamoto, T., Y. Sato, T. Yamazaki, and A. Hayashi, Clinically mild encephalitis/encephalopathy with a reversible splenial lesion associated with febrile urinary tract infection. Eur J Pediatr, 2014. 173(4): p. 533-6.
216. Okumura, A., et al., Transiently reduced water diffusion in the corpus callosum in infants with benign partial epilepsy in infancy. Brain Dev, 2010. 32(7): p. 564-6.
217. Osuka, S., et al., Mild encephalitis/encephalopathy with a reversible splenial lesion: evaluation by diffusion tensor imaging. Two case reports. Neurol Med Chir (Tokyo), 2010. 50(12): p. 1118-22.
218. Ozenen, G.G., et al., The first pediatric case of mild encephalitis/encephalopathy with a reversible splenial lesion (MERS) associated with Neisseria meningitidis. Brain Dev, 2021.
219. Oztoprak, I., et al., Transient splenial lesions of the corpus callosum in different stages of evolution. Clin Radiol, 2007. 62(9): p. 907-13.
220. Palabiyik, F., et al., Imaging of Multisystem Inflammatory Disease in Children (MIS-C) Associated With COVID-19. Acad Radiol, 2021. 28(9): p. 1200-1208.
221. Pan, J.J., et al., Mild encephalitis/encephalopathy with a reversible splenial lesion: five cases and a literature review. Neurol Sci, 2015. 36(11): p. 2043-51.
222. Panciani, P.P., E. Roca, G. Lodoli, and M.M. Fontanella, Sudden worsening after subdural haematoma surgery: will there be a corpus callosum injury? BMJ Case Rep, 2014. 2014.
223. Parikh, N.C. and M. Kulkarni, Transient and reversible focal lesion involving the splenium of the corpus callosum in a person with epilepsy. Ann Indian Acad Neurol, 2008. 11(2): p. 123-4.
224. Park, J.H., et al., A Case of Scrub Typhus Related Encephalopathy Presenting as Rapidly Progressive Dementia. Dement Neurocogn Disord, 2017. 16(3): p. 83-86.
225. Peng, Q., Q. You, J. Zhang, and S. Liu, Isolated involvement of corpus callosum in metronidazole-induced encephalopathy with concomitant peripheral neuropathy: A case report. Medicine (Baltimore), 2020. 99(20): p. e20198.
226. Perrain, V., et al., Leukoencephalopathy with transient splenial lesions related to 5-fluorouracil or capecitabine. Eur J Neurol, 2021. 28(7): p. 2396-2402.

227. Polster, T., M. Hoppe, and A. Ebner, Transient lesion in the splenium of the corpus callosum: three further cases in epileptic patients and a pathophysiological hypothesis. J Neurol Neurosurg Psychiatry, 2001. 70(4): p. 459-63.
228. Prilipko, O., J. Delavelle, F. Lazeyras, and M. Seeck, Reversible cytotoxic edema in the splenium of the corpus callosum related to antiepileptic treatment: report of two cases and literature review. Epilepsia, 2005. 46(10): p. 1633-6.
229. Qing, Y., et al., Statistical Analysis of the Apparent Diffusion Coefficient in Patients with Clinically Mild Encephalitis/Encephalopathy with a Reversible Splenial Lesion Indicates That the Pathology Extends Well beyond the Visible Lesions. Magn Reson Med Sci, 2020. 19(1): p. 14-20.
230. Renard, D., A. Bonafe, and C. Heroum, Transient lesion in the splenium of the corpus callosum after oral corticoid therapy. Eur J Neurol, 2007. 14(8): p. e19-20.
231. Renard, D., et al., Mild encephalitis/encephalopathy with a reversible splenial, white matter, putaminal, and thalamic lesions following anti-Yo rhombencephalitis. Acta Neurol Belg, 2012. 112(4): p. 405-7.
232. Rolshoven, J., K. Fellows, R. Ania, and B.J. Tabaac, Vertigo and Cytotoxic Lesions of the Corpus Callosum: Report with Review of the Literature. Case Rep Neurol Med, 2021. 2021: p. 5573822.
233. Rozen, T.D. and H.A. Robles, A Reversible Cytotoxic Lesion of the Corpus Callosum Developing after a Rapid Alteration in Cerebrospinal Fluid Pressure/Volume in a Patient with New Daily Persistent Headache. Case Rep Neurol Med, 2020. 2020: p. 8849645.
234. Ruscheweyh, R., M. Marziniak, and S. Evers, Reversible focal splenial lesions in facial pain patients treated with antiepileptic drugs: case report and review of the literature. Cephalalgia, 2009. 29(5): p. 587-90.
235. Ryu, H.U., J.Y. Chung, B.S. Shin, and H.G. Kang, Lithium induced reversible Splenial lesion in neuroleptic malignant syndrome like symptoms: two case reports. BMC Neurol, 2020. 20(1): p. 164.
236. Sadohara, M., T. Arai, and K. Matsuura, Clinically mild encephalitis/encephalopathy with reversible splenial lesion (MERS) associated with Mycoplasma pneumoniae pneumonia: An adult case and review of the literature. Clin Case Rep, 2020. 8(12): p. 2955-2961.
237. Sáenz-Farret, M., et al., The Spectrum of Acute Disseminated Encephalomyelitis and Mild Encephalopathy with Reversible Splenial Lesion. Case Rep Neurol Med, 2019. 2019: p. 9272074.
238. Saif, H., A. Valavanis, and D. Johnson, A rare case of reversible splenial lesion in third trimester. Radiol Case Rep, 2021. 16(10): p. 3081-3083.
239. Samanta, D., Transient lesion in the splenium of the corpus callosum in status migrainosus. Acta Neurol Belg, 2015. 115(3): p. 397-8.
240. Sano, F., et al., Clinically Mild Encephalopathy With a Reversible Splenial Lesion Type 2 Caused by Human Herpesvirus 6 Infection. Pediatr Neurol, 2020. 113: p. 43-45.

241. Sathananthasarma, P., P.N. Weeratunga, and T. Chang, Reversible splenial lesion syndrome associated with dengue fever: a case report. BMC Res Notes, 2018. 11(1): p. 412.

242. Sato, T., et al., Kawasaki disease-associated MERS: pathological insights from SPECT findings. Brain Dev, 2012. 34(7): p. 605-8.

243. Sawagashira, R., et al., Transient lesions of the splenium of the corpus callosum following rapid withdrawal of levetiracetam. Epileptic Disord, 2017. 19(3): p. 379-382.

244. Schubert, L., et al., Pandemic-related delay of falciparum malaria diagnosis in a traveler leading to cerebral malaria. J Travel Med, 2021.

245. Sekine, T., et al., Transient splenial lesion after recovery of cerebral vasoconstriction and posterior reversible encephalopathy syndrome: a case report of eclampsia. Intern Med, 2012. 51(11): p. 1407-11.

246. Sen, K., G. Guha, K. Khandelwal, and J. Lalhmachhuana, The enigma of transient splenial hyperintensity: In cryptococcal meningitis. J Neurosci Rural Pract, 2013. 4(3): p. 352-5.

247. Shah, S., A. Keil, K. Gara, and L. Nagarajan, Neurologic complications of influenza. J Child Neurol, 2014. 29(9): p. Np49-53.

248. Shankar, B., R. Narayanan, P. Muralitharan, and B. Ulaganathan, Evaluation of mild encephalitis/encephalopathy with a reversible splenial lesion (MERS) by diffusion-weighted and diffusion tensor imaging. BMJ Case Rep, 2014. 2014.

249. Sharma, B., et al., Transient elevation of cerebrospinal fluid protein in a patient of mild encephalitis with reversible lesion in the splenium: a case report. Malays J Med Sci, 2014. 21(3): p. 94-7.

250. Shi, B.C., et al., Mild encephalitis/encephalopathy with a reversible splenial lesion secondary to encephalitis complicated by hyponatremia: A case report and literature review. Medicine (Baltimore), 2019. 98(47): p. e17982.

251. Shimbo, A., et al., Complications of Listeria meningitis in two immunocompetent children. Pediatr Int, 2018. 60(5): p. 491-492.

252. Shimizu, H., et al., Extensive neuroimaging of a transient lesion in the splenium of the corpus callosum. Eur J Neurol, 2007. 14(1): p. e37-9.

253. Shimono, H., et al., A rare etiology of mild encephalitis/encephalopathy with reversible splenial lesion. Clin Case Rep, 2021. 9(9): p. e04759.

254. Singh, P., D. Gogoi, S. Vyas, and N. Khandelwal, Transient splenial lesion: Further experience with two cases. Indian J Radiol Imaging, 2010. 20(4): p. 254-7.

255. Smail, R.C., J. Baird-Gunning, J. Drummond, and K. Ng, A case report of a transient splenial lesion related to HaNDL syndrome. Cephalalgia, 2020. 40(10): p. 1119-1122.

256. Soma, N., Y. Aizawa, M. Matsunaga, and A. Saitoh, Clinically Mild Encephalitis/Encephalopathy with a Reversible Splenial Lesion Associated with Rhinovirus. Pediatr Infect Dis J, 2021. 40(3): p. e122-e125.

257. Soon, G.S., et al., Reversible splenial lesion syndrome in pediatric systemic lupus erythematosus. J Rheumatol, 2012. 39(8): p. 1698-9.
258. Sun, D., et al., Mild encephalopathy/encephalitis with a reversible splenial lesion (MERS): A report of five neonatal cases. J Huazhong Univ Sci Technolog Med Sci, 2017. 37(3): p. 433-438.
259. Suzuki, H., T. Kusaka, and H. Okada, Clinically mild encephalitis/encephalopathy with a reversible splenial lesion caused by human parvovirus b19 infection: a case of two brothers with hereditary spherocytosis. Pediatr Neurol, 2014. 51(3): p. 470-2.
260. Tada, H., et al., Clinically mild encephalitis/encephalopathy with a reversible splenial lesion. Neurology, 2004. 63(10): p. 1854-8.
261. Tahara, J., et al., Mild Encephalopathy with Reversible Lesions in the Splenium of Corpus Callosum and Bilateral Cerebral Deep White Matter in Identical Twins. Pediatr Rep, 2016. 8(3): p. 6615.
262. Takahashi, I., H. Yano, and M. Kinjo, Mild encephalitis/encephalopathy with a reversible splenial lesion (MERS) following influenza virus infection. BMJ Case Rep, 2020. 13(5).
263. Takahashi, K., et al., A case of primary erythermalgia, wintry hypothermia and encephalopathy. Neuropediatrics, 2007. 38(3): p. 157-9.
264. Takahashi, Y., et al., Reversible splenial lesion in postpartum cerebral angiopathy: a case report. J Neuroimaging, 2014. 24(3): p. 292-4.
265. Takanashi, J., et al., Widening spectrum of a reversible splenial lesion with transiently reduced diffusion. AJNR Am J Neuroradiol, 2006. 27(4): p. 836-8.
266. Takanashi, J., A. Imamura, F. Hayakawa, and H. Terada, Differences in the time course of splenial and white matter lesions in clinically mild encephalitis/encephalopathy with a reversible splenial lesion (MERS). J Neurol Sci, 2010. 292(1-2): p. 24-7.
267. Takanashi, J., et al., Clinical and radiological features of rotavirus cerebellitis. AJNR Am J Neuroradiol, 2010. 31(9): p. 1591-5.
268. Takanashi, J., et al., Clinically mild encephalitis with a reversible splenial lesion (MERS) after mumps vaccination. J Neurol Sci, 2015. 349(1-2): p. 226-8.
269. Takanashi, J., et al., Kawasaki disease complicated by mild encephalopathy with a reversible splenial lesion (MERS). J Neurol Sci, 2012. 315(1-2): p. 167-9.
270. Takanashi, J., H. Tada, H. Kuroki, and A.J. Barkovich, Delirious behavior in influenza is associated with a reversible splenial lesion. Brain Dev, 2009. 31(6): p. 423-6.
271. Takanashi, J., et al., Encephalopathy with a reversible splenial lesion is associated with hyponatremia. Brain Dev, 2009. 31(3): p. 217-20.
272. Takatsu, H., N. Ishimaru, M. Ito, and S. Kinami, Mild Encephalitis/Encephalopathy with a Reversible Splenial Lesion in an Adult Patient with Influenza. Intern Med, 2017. 56(22): p. 3093-3095.

273. Takayama, H., M. Kobayashi, M. Sugishita, and B. Mihara, Diffusion-weighted imaging demonstrates transient cytotoxic edema involving the corpus callosum in a patient with diffuse brain injury. Clin Neurol Neurosurg, 2000. 102(3): p. 135-9.
274. Takayanagi, M., et al., Kleine-Levin syndrome elicited by encephalopathy with reversible splenial lesion. Pediatr Int, 2017. 59(8): p. 929-931.
275. Takeuchi, S., Y. Takasato, and H. Masaoka, Epstein-Barr virus encephalitis with a reversible splenial lesion. Intern Med, 2012. 51(3): p. 341-2.
276. Tang, Y., et al., Clinical and imaging features of reversible splenial lesion syndrome with language disorder. Transl Neurosci, 2020. 11(1): p. 210-214.
277. Tani, M., et al., Isolated reversible splenial lesion in adult meningitis: a case report and review of the literature. Intern Med, 2007. 46(18): p. 1597-600.
278. Tascilar, N., et al., Unusual combination of reversible splenial lesion and meningitis-retention syndrome in aseptic meningomyelitis. Clinics (Sao Paulo), 2009. 64(9): p. 932-7.
279. Tetsuka, S., T. Kamimura, G. Ohki, and R. Hashimoto, Reversible lesion in the splenium of the corpus callosum in a patient with chronic alcoholism. J Gen Fam Med, 2020. 21(3): p. 84-86.
280. Theeler, B.J., D.J. Wilson, C.M. Crawford, and M. Grazko, Optic neuropathy and a reversible splenial lesion after gastric bypass: shared pathophysiology? J Neurol Sci, 2010. 291(1-2): p. 92-4.
281. Toi, H., et al., Clinical Features of Cytotoxic Lesions of the Corpus Callosum Associated with Aneurysmal Subarachnoid Hemorrhage. AJNR Am J Neuroradiol, 2021. 42(6): p. 1046-1051.
282. Tomizawa, Y., et al., Diagnostic Utility of Splenial Lesions in a Case of Legionnaires' Disease due to Legionella pneumophila Serogroup 2. Intern Med, 2015. 54(23): p. 3079-82.
283. Touat, M., et al., Encephalopathy associated with a reversible splenial lesion in riboflavin-responsive multiple acyl-CoA dehydrogenase deficiency. Rev Neurol (Paris), 2018. 174(10): p. 747-750.
284. Tsitsikas, D.A., et al., Complete neurological recovery from fat embolism syndrome in sickle cell disease after sequential red cell exchange transfusion and therapeutic plasma exchange. Transfus Apher Sci, 2021: p. 103226.
285. Tsubouchi, Y., et al., Use of high b value diffusion-weighted magnetic resonance imaging in acute encephalopathy/encephalitis during childhood. Brain Dev, 2018. 40(2): p. 116-125.
286. Tsuji, M., et al., Cytotoxic lesion of the corpus callosum exclusively at the genu in a case of callosal hypogenesis. J Neuroradiol, 2019. 46(3): p. 222-223.
287. Tsuji, M., T. Yoshida, C. Miyakoshi, and T. Haruta, Is a reversible splenial lesion a sign of encephalopathy? Pediatr Neurol, 2009. 41(2): p. 143-5.
288. Tuscano, A., et al., Transient blindness associated with mild encephalitis/encephalopathy with a reversible splenial lesion (MERS): a case report and review of literature. Ital J Pediatr, 2020. 46(1): p. 152.

289. Uchigami, H., et al., Spontaneous Intracranial Hypotension with a Reversible Splenial Lesion after Swimming. Intern Med, 2020. 59(20): p. 2593-2596.
290. Udaya, S.C., B.N. Chauhan, and V.J. Philip, Bright splenium of a psychotic mind. Ann Indian Acad Neurol, 2015. 18(1): p. 80-3.
291. Ueda, F., et al., Splenial and white matter lesions showing transiently-reduced diffusion in mild encephalopathy monitored with MR spectroscopy and imaging. Magn Reson Med Sci, 2014. 13(4): p. 271-5.
292. Ueda, N., S. Minami, and M. Akimoto, Mycoplasma pneumoniae-associated mild encephalitis/encephalopathy with a reversible splenial lesion: report of two pediatric cases and a comprehensive literature review. BMC Infect Dis, 2016. 16(1): p. 671.
293. Uygur Kucukseymen, E., et al., Reversible Splenial Lesion Syndrome After Intravenous Immunoglobulin Treatment for Guillain-Barre Syndrome. Clin Neuropharmacol, 2017. 40(5): p. 224-225.
294. Vanderschueren, G., K. Schotsmans, E. Maréchal, and R. Crols, Mild encephalitis with reversible splenial (MERS) lesion syndrome due to influenza B virus. Pract Neurol, 2018. 18(5): p. 391-392.
295. Ventresca, S., et al., Clinically Mild Encephalopathy with a Reversible Splenial Lesion Caused by Influenza B Virus in an Unvaccinated Child. Pediatr Rep, 2021. 13(1): p. 72-75.
296. Vollmann, H., et al., Isolated reversible splenial lesion in tick-borne encephalitis: a case report and literature review. Clin Neurol Neurosurg, 2011. 113(5): p. 430-3.
297. Wang, J., E. Stewart, K. Dapaah-Afriyie, and A. Finn, Mild encephalopathy with reversible splenial lesion in a patient with influenza A infection--first report in an adult patient in the USA. BMJ Case Rep, 2015. 2015.
298. Watanabe, T., et al., Transient renal dysfunction with reversible splenial lesion. Pediatr Int, 2014. 56(5): p. e68-71.
299. Winslow, H., B. Mickey, and E.M. Frohman, Sympathomimetic-induced kaleidoscopic visual illusion associated with a reversible splenium lesion. Arch Neurol, 2006. 63(1): p. 135-7.
300. Xu, J., et al., Mild encephalitis/encephalopathy with a reversible splenial lesion (MERS) associated with bacteria meningitis caused by listeria monocytogenes: A case report. Medicine (Baltimore), 2018. 97(30): p. e11561.
301. Xu, W., et al., Clinical and Magnetic Resonance Imaging Features of Reversible Splenial Lesion Syndrome in Adults: A Small Case Series. Eur Neurol, 2019. 82(4-6): p. 86-92.
302. Xu, Z., et al., Reversible splenial lesion syndrome with mental disorders as only manifestation. BMC Neurol, 2021. 21(1): p. 356.
303. Xue, J., et al., A cohort study of mild encephalitis/encephalopathy with a reversible splenial lesion in children. Brain Behav, 2021. 11(8): p. e2306.

304. Yaguchi, M., H. Yaguchi, T. Itoh, and K. Okamoto, Encephalopathy with isolated reversible splenial lesion of the corpus callosum. Intern Med, 2005. 44(12): p. 1291-4.

305. Yamaguchi, Y., et al., Transient Lesion of the Splenium of the Corpus Callosum after Acute Ischemic Stroke. Intern Med, 2019. 58(7): p. 1011-1015.

306. Yamashita, C., et al., Transient interhemispheric disconnection in a case of insulinoma-induced hypoglycemic encephalopathy. J Neurol Sci, 2013. 335(1-2): p. 233-7.

307. Yamashita, T., S. Tokushige, R. Maekawa, and Y. Shiio, Reversible splenial lesion associated with acute HIV infection. Intern Med, 2012. 51(12): p. 1643.

308. Yang, J., et al., Reversible splenial lesion syndrome (RESLES) due to acute intermittent porphyria with a novel mutation in the hydroxymethylbilane synthase gene. Orphanet J Rare Dis, 2020. 15(1): p. 98.

309. Yang, J., et al., Reversible splenial lesion syndrome in sisters with sensorineural deafness as the first manifestation. Heliyon, 2021. 7(5): p. e07057.

310. Yang, Q., C.C. Chang, M. Liu, and Y.Q. Yu, Sequential occurrence of eclampsia-associated posterior reversible encephalopathy syndrome and reversible splenial lesion syndrome (a case report): proposal of a novel pathogenesis for reversible splenial lesion syndrome. BMC Med Imaging, 2019. 19(1): p. 35.

311. Yeh, I.B., L.C. Tan, and Y.Y. Sitoh, Reversible splenial lesion in clinically mild encephalitis. Singapore Med J, 2005. 46(12): p. 726-30.

312. Yeom, J.S., et al., Mild encephalopathy with a reversible splenial lesion in a girl with acute pyelonephritis. Korean J Pediatr, 2018. 61(2): p. 64-67.

313. Yıldız, A.E., et al., Mild encephalitis/encephalopathy with a reversible splenial lesion in children. Diagn Interv Radiol, 2018. 24(2): p. 108-112.

314. Yokoyama, T., S. Yamada, N. Doichi, and E. Kato, Rotavirus-infected children with clinically mild encephalopathy with a reversible splenial lesion (MERS). BMJ Case Rep, 2013. 2013.

315. Youn, T. and H. Yang, Cytotoxic Lesion of the Corpus Callosum (CLOCCs) after SARS-CoV-2 mRNA Vaccination. J Korean Med Sci, 2021. 36(31): p. e228.

316. Yuan, J., et al., Mild encephalitis/encephalopathy with reversible splenial lesion (MERS) in adults-a case report and literature review. BMC Neurol, 2017. 17(1): p. 103.

317. Yuan, Z.F., et al., Clinically mild encephalitis/encephalopathy with a reversible splenial lesion associated with Mycoplasma pneumoniae infection. BMC Infect Dis, 2016. 16: p. 230.

318. Zhang, S., J. Feng, and Y. Shi, Transient widespread cortical and splenial lesions in acute encephalitis/encephalopathy associated with primary Epstein-Barr virus infection. Int J Infect Dis, 2016. 42: p. 7-10.

319. Zhang, S., Y. Ma, and J. Feng, Clinicoradiological spectrum of reversible splenial lesion syndrome (RESLES) in adults: a retrospective study of a rare entity. Medicine (Baltimore), 2015. 94(6): p. e512.

320. Zhang, X., et al., Reversible splenial lesion syndrome in children: clinical analysis and summary of a case series. J Int Med Res, 2020. 48(4): p. 300060520914202.
321. Zhang, Y. and Q. Shi, A Wide Range of High Signal Intensities on Brain Image in Adult Mycoplasma Pneumoniae-Associated Mild Encephalitis/Encephalopathy with a Reversible Splenial Lesion. Neurol India, 2021. 69(4): p. 1112-1113.
322. Zhang, Z., et al., Mild Encephalitis/Encephalopathy with a Reversible Isolated Splenial Lesion (MERS) in Adult Patients: A Small Case Series. Eur Neurol, 2020. 83(3): p. 279-286.
323. Zhu, Y., et al., Reversible splenial lesion syndrome associated with encephalitis/encephalopathy presenting with great clinical heterogeneity. BMC Neurol, 2016. 16: p. 49.
324. Zhuang, B., et al., The assessment of mild encephalopathy with a reversible splenial lesion (MERS) using high b-value DWI. Medicine (Baltimore), 2019. 98(44): p. e17638.
